# The burden of multimorbidity-associated acute hospital admissions in Malawi and Tanzania: a prospective multicentre cohort study

**DOI:** 10.1016/S2214-109X(25)00113-5

**Published:** 2025-06-25

**Authors:** Stephen A Spencer, Nateiya M Yongolo, Ibrahim G Simiyu, Hendry R Sawe, Paul Dark, Stephen B Gordon, Matthew P Rubach, Rachel Manongi, Julian T Hertz, Gimbo Hyuha, Grasiana Kimario, Juma Mfinanga, Blandina T Mmbaga, Adamson S Muula, Mulinda Nyirenda, Jacob Phulusa, Laura Rosu, Alice H Rutta, Francis Sakita, Charity Salima, Miriam Taegtmeyer, Sarah Urasa, Sarah A White, Jamie Rylance, Felix Limbani, Eve Worrall, Ben Morton, Multilink Consortium, Sanjura Biswaro, Yesse Bumija, Robert Chuwa, Rose Freddy, Mwamini Kacheuka, Frank Kimaro, Zanuni Kweka, Rachel Mangoni, Martha Oshoseni, Philoteus Sakasaka, Constantine Tarimo, Gidion Tesha, Safina Baleche, Yusuph Chimpaye, Frank Gugu, Naftari Mahimbo, Ramadhani Mashoka, Vicky Mlele, Hussein Abdallah Moremi, Noela Mpili, Herieth Cliff Mushi, Nsajigwa Mwakyambiki, Benjamin Paulo Mwenda, Abdulaziz Abdallah Nassoro, Nuhu Richard, Chiku Simbano, Marlen Chewani, Beatrice Chinoko, Marc Henrion, Sylvester Kaimba, Maureen Kandiero, Lucy Keyala, Florence Malowa, Peter Mandala, Mercy Mkandawire, Matthew Mlongoti, Bright Mnesa, Albert Mukatipa, Alfred Muyaya, Deborah Nyirenda, Jacob Phulusa, Diana Msindira, Genesis Chowe, Nicola Desmond, Firdaus Hafidz, Amy Smith, Yusuf Iqbal, Joanna Jozefiak, Marlen Chawani, Rhona Mijumbi, Treighcy G Banda, Sangwani Salimu, Augustine Choko

**Affiliations:** a Malawi–Liverpool–Wellcome Programme, Blantyre, Malawi; b Liverpool School of Tropical Medicine, Liverpool, UK; c Queen Elizabeth Central Hospital, Blantyre, Malawi; d Kilimanjaro Clinical Research Institute, Moshi, Tanzania; e KCMC University, Moshi, Tanzania; f Muhimbili University of Health and Allied Sciences, Dar es Salaam, Tanzania; g Humanitarian and Conflict Response Institute, University of Manchester, Manchester, UK; h Duke University School of Medicine, Durham, NC, USA; i The Kamuzu University of Health Sciences, Blantyre, Malawi; j Kilimanjaro Christian Medical Centre, Moshi, Tanzania; k Achikondi Women Community Clinic, Lilongwe, Malawi

## Abstract

**Background:**

The global burden of multimorbidity—the coexistence of two or more long-term conditions—is increasing. Limited access to primary care in sub-Saharan Africa means acute hospital admission is often the sentinel multimorbidity presentation. This prospective multicentre cohort study aimed to describe the burden, constituent diseases, and outcomes of multimorbidity among patients acutely admitted to hospital in Malawi and Tanzania.

**Methods:**

Adults (ie, those aged ≥18 years) admitted to four hospitals (two tertiary and two district hospitals) with acute medical conditions were consecutively recruited within 24 h of presentation and followed up for 90 days. We estimated the prevalence of HIV infection, diabetes, hypertension, and chronic kidney disease using commercially available point-of-care tests, and captured self-reported and clinical diagnoses (n/N [%]). Health economic data were summarised by median and IQR and modelled using generalised linear models. All-cause 90-day mortality was summarised with Kalplan–Meier plots and analysed using Cox regression models.

**Findings:**

1407 adults (657 [46·7%] were female and 750 [53·3%] were male; mean age was 52·3 years [SD 18·4]) were recruited. We examined multimorbidity prevalence in 1007 participants admitted to three hospitals that accept admissions directly from the community. Multimorbidity was found in 473 (47·0%) of 1007 participants and 292 (29·0%) had a single long-term condition. Outcomes at 90 days were determined for 1317 (93·6%) of 1407 participants. Adjusted 90-day mortality was higher in participants with multimorbidity (335 [41·7%] of 804; hazard ratio 1·5 [95% CI 1·1–2·1]) and those with one long-term condition (80 [28·3%] of 283; 1·5 [1·0–2·1]); compared with those with no long-term conditions (31 [13·5%] of 230). Health-related quality of life was lower in participants with multimorbidity compared with those with one long-term condition (median 0·402 [IQR –0·037 to 0·644] *vs* 0·557 [0·140 to 0·730]; p=0·005) at baseline, and at final observation (0·858 [0·667 to 1·00] *vs* 1·00 [0·589 to 1·00] respectively; p=0·01). In Tanzania, medical costs incurred by patients were higher in participants with multimorbidity compared with those with one long-term condition (relative effect 5·77 [95% CI 2·99–11·15]; p<0·0001).

**Interpretation:**

Multimorbidity is common in patients admitted to hospital in Malawi and Tanzania and associated with worse survival and increased cost. Multimorbidity is an urgent public health threat that requires fundamental health-care delivery reform to address population needs.

**Funding:**

National Institute for Health and Care Research and Wellcome Trust.

**Translations:**

For the Chichewa and Kiswahili translations of the abstract see Supplementary Materials section.

## Introduction

The global burden of multimorbidity (defined as the coexistence of two or more long-term conditions)^1^ is rising, largely driven by ageing populations. In sub-Saharan Africa, this challenge is compounded by the high prevalence of both chronic non-communicable diseases (NCDs) and communicable diseases, including HIV. In these settings, delayed diagnosis and acute presentation of decompensated chronic disease to hospital are common.^2^, ^3^ This in turn puts additional strain on health-care systems, in which recognition of multimorbidity is constrained by resource limitations and vertical health-care delivery models.

Most multimorbidity data come from high-income countries in which there are higher hospitalisation and mortality rates among patients with multimorbidity compared with patients without multimorbidity, particularly in older adults (ie, those aged 65 years and older).^4^ Multimorbidity data from sub-Saharan Africa are predominantly limited to community-based studies.^5^ In this region, multimorbidity occurs in the context of epidemic HIV infection compounded by an increasing burden of NCDs.^6^ Interaction between HIV and its required treatments leads to earlier onset and progression of NCDs.^7^ Limited access to primary care, diagnostics, and treatment impedes disease management, increasing downstream end-organ disease, morbidity, hospitalisation, and mortality.^8^ Studies highlight high rates of HIV, diabetes, and hypertension in hospital settings in Africa, in which undiagnosed or uncontrolled disease is common.^2^ Hospital care therefore has a crucial role in multimorbidity diagnosis, initial management, and linkage to medium-term and long-term community-based care. Furthermore, measures are required to mitigate against high out-of-pocket expenditure for both medical and non-medical-related costs and to reduce the impact of multimorbidity on health-related quality of life (HRQoL).^9^, ^10^

**Research in context**

**Evidence before this study**

We conducted a systematic review and meta-analysis that has previously been published. On May 12, 2023, we searched MEDLINE, Embase, Global Index Medicus, Global Health, and SciELO with no language restrictions for publications published between Jan 1, 2010, and May 12, 2023. Our search terms were: (“multimorbidity” OR “hypertension” OR “diabetes” OR “HIV/AIDS” OR “kidney dysfunction” OR “hypercholesterolaemia” OR “cerebrovascular event” OR “ischaemic heart disease” OR “chronic liver disease” OR “heart failure” OR “chronic kidney disease” OR “chronic obstructive pulmonary disease” OR “obesity” OR “alcohol use” OR “tobacco”) AND “sub-Saharan Africa” AND “acute hospital care” AND “adults”. We updated our search on March 5, 2025, on MEDLINE and found no studies had reported on prevalence or disease constituents of multimorbidity in sub-Saharan African hospitals. Published pooled estimates for single chronic diseases among medical admissions show high prevalence of HIV infection (36·4%; 95% CI 31·3–41·8); hypertension (24·4%; 16·7–34·2); diabetes (11·9%; 9·9–14·3); heart failure (8·2%; 5·6–11·9); chronic kidney disease (7·7%; 3·9–14·7); and cerebrovascular event (6·8%; 4·7–9·6). We observed that existing literature applied heterogeneous diagnostic criteria and case definitions, increasing uncertainty around these estimates.

Studies from high-income countries report an association between multimorbidity and health-care costs in older adults (ie, those aged 65 years and older), with some evidence that provider costs and out-of-pocket costs to patients increase exponentially with the number of long-term conditions. We also searched PubMed for published articles between Jan 1, 2010, and April 15, 2023 (search updated on March 5, 2025) using key search terms (“multimorbidity”) AND (“healthcare expenditure”) AND (“health-related quality of life”) AND (“sub-Saharan Africa”). Although many studies explored the costs associated with single long-term conditions, we found no studies reporting on patient or health system costs associated with multimorbidity in sub-Saharan Africa. Health-related quality of life has been shown to be negatively associated with multimorbidity in countries of all income strata.

**Added value of this study**

Our multicentre prospective cohort study describes the burden, constituent diseases, health-related quality of life, and outcomes of multimorbidity among patients admitted to hospital in Malawi and Tanzania with acute medical conditions; and measures patient cost and effect on incomes among a subset of participants. We report a high prevalence of multimorbidity (473 [47·0%] of 1007); high rates of disability (593 [41·1%]) and frailty (746 [39·9%]); and high 90-day mortality among patients with either one long-term condition (80 [28·3%] of 283) or multimorbidity (335 [41·7%] of 804). We also describe disease constituents of multimorbidity, most commonly comprising hypertension, diabetes, and HIV. The economic burden (patient cost) and impact (health-related quality of life, income loss, and catastrophic health expenditure) are substantial for patients with acute illness in Malawi and Tanzania, with higher medical costs observed in individuals with multimorbidity in Tanzania.

**Implications of all the available evidence**

Health-care delivery models focus on a single presenting complaint and typically do not systematically screen for or treat long-term conditions. We observed poor disease control, particularly for non-communicable diseases. We observed poor health-related quality of life, high costs, and income reductions among acutely admitted patients, particularly for those with multimorbidity. Further research is urgently required to test context-sensitive models of health systems delivery to identify and control chronic disease; prevent complications; reduce disability and mortality; and ensure financial protection for patients. Building on the successful roll-out of protocolised vertical programmes of care for people living with HIV infection, we recommend the development and evaluation of standardised models of care to address the rising rates of multimorbidity in this population.

To the best of our knowledge, no studies have investigated prevalence, disease constituents, and health and economic outcomes for patients with multimorbidity in hospital settings in sub-Saharan Africa. Such data are vital to prioritise health-service delivery, and to effectively design, finance, and deliver patient-centred care models. We hypothesised that there would be high prevalence of multimorbidity in hospitalised patients and that multimorbidity would be associated with increased mortality, increased costs, and lower HRQoL in Africa.

## Methods

### Study design

This study was conducted according to our a priori published study protocol^11^ and reported using STROBE guidelines (appendix 3 p 3). This prospective cohort study was conducted in four hospitals in Malawi and Tanzania, including two tertiary (national referral) hospitals (ie, the Queen Elizabeth Central Hospital [1350 bed capacity], Blantyre, Malawi; and the Muhimbili National Hospital [1500 bed capacity], Dar es Salaam, Tanzania) and two district hospitals (Chiradzulu District Hospital [300 bed capacity], Chiradzulu, Malawi; and the Hai District Hospital [140 bed capacity], Hai District, Tanzania). Site descriptions are provided in appendix 3 (p 4). Recruitment commenced on Sept 20, 2022. 90-day follow-up for all participants was completed on March 28, 2024.

### Participants

Eligible participants were adults (ie, those aged ≥18 years) admitted to hospital with an acute medical condition (full eligibility criteria are provided in the study protocol).^11^ Participants were screened for eligibility at hospital admission and consecutively recruited within 24 h of presentation. Participants were followed up during their hospital admission (ie, on day 0, 2, 5, 7 and at discharge), by telephone at day 30, and in person at day 90. Health economic data were collected for one in three randomly selected participants (via a computer generated code, incorporated within the electronic case report form); HRQoL were collected from all participants. All study procedures were conducted by research staff.

Ethical approvals were obtained from the Liverpool School of Tropical Medicine, Liverpool, UK (21-086); the College of Medicine Research and Ethics Committee, Blantyre, Malawi (P·11/21/3462); the National Institute for Medical Research (NIMR), Dar es Salaam**,** Tanzania (NIMR/HQ/R·8a/Vol·IX/4008); and the Kilimanjaro Christian Medical Centre, Moshi**,** Tanzania (2570). Written informed consent (or consultee assent for individuals who did not have capacity) was obtained for all participants. Full details of our consent processes have been published previously.^11^

### Procedures

Participants were systematically screened for multimorbidity with a focus on common, pre-selected conditions identified from our systematic review.^2^ We defined multimorbidity as two or more long-term conditions, including primary conditions (such as HIV infection, hypertension, diabetes, chronic kidney disease, and depression) and secondary conditions (such as heart failure, ischaemic heart disease, cerebrovascular events, chronic liver disease, and chronic obstructive pulmonary disease). We used Conformité Européenne-marked, commercially available point-of-care tools to identify HIV infection, hypertension, diabetes, and chronic kidney disease. Primary conditions were diagnosed using internationally accepted criteria, including WHO's package of essential non-communicable disease interventions^12^ (hypertension and diabetes); WHO Consolidated Guidelines on HIV Testing Services;^13^ and the Kidney Disease: Improving Global Outcomes guidelines,^14^ using the 2021 Chronic Kidney Disease Epidemiology Collaboration race-free calculation to estimate glomerular filtration rate. Manufacturer instructions were followed for calibration and quality assurance (details are presented in the study protocol).^11^ We used the Patient Health Questionnaire (PHQ-2 and PHQ-9) screening tools for depression (defined as a PHQ-9 score of ≥10).^15^ Detailed case definitions are available in appendix 3 (pp 5–6). In instances of incomplete data, our diagnostic pathways (appendix 3 pp 7–12) incorporated all relevant data (ie, self-reported diagnoses and clinically recorded diagnoses and medical treatments). The research staff did not participate in clinical management but made test results and routine physiological data available to treating clinicians. Secondary condition data were captured from patient reports and clinical records. We collected functional outcomes (evidence to underpin outcome selection is available in appendix 3 p 12), including: frailty (clinical frailty scale; defined as a score of ≥5);^16^ disability (Washington Group-Short Set on Functioning; defined as at least a lot of difficulty in one or more domains);^17^ and HRQoL, using the EuroQol EQ-5D-5L in Chichewa (Malawi) or Kiswahili (Tanzania). We used the Uganda value set^18^ (geographically closest, as no value sets are available for Malawi or Tanzania), to convert EQ-5D-5L results to HRQoL utility scores. We used a modified tuberculosis patient cost tool^19^ to collect data on medical costs (eg, drugs, medical supplies, tests, and remedies), non-medical costs (eg, food and transport), and indirect costs (eg, income and work days lost).

### Outcomes

The primary objective was to determine the prevalence of multimorbidity in adults admitted to hospital with acute medical conditions in Malawi and Tanzania. We calculated prevalence from both hospitals in Malawi (ie, the Queen Elizabeth Central Hospital and Chiradzulu District Hospital), and the Hai District Hospital in Tanzania, which receives admissions directly from the community. Data from Muhimbili National Hospital in Tanzania was not used for prevalence outcomes as this tertiary centre accepts admissions predominantly referred from hospitals and would bias prevalence estimates (appendix 3 pp 17–21). Secondary clinical objectives were to determine the prevalence of individual long-term diseases; disease control (criteria available in appendix 3 pp 5–6); hospital re-admission; and survival at 30 days and 90 days. The Muhimbili National Hospital site includes tertiary care patients, admitted from secondary care hospitals with increased prevalence of multimorbidity. Therefore, this population was not used to estimate mulitmorbidity prevalence in our cohort but was used within the outcome data analysis.

The primary health economic objective was to compare HRQoL health utility (indexed EQ-5D-5L) between participants with zero, one, and two or more long-term conditions. Secondary objectives were to compare direct and indirect costs; income reduction pre-hospitalisation and post-hospitalisation; and catastrophic cost incursions between these groups. Direct costs comprised total medical and non-medical expenses from pre-admission care, inpatient stay, and up to death or end of follow-up. Indirect costs were calculated as workdays lost (over the cost measurement period) multiplied by the reported mean monthly pre-illness income, divided by 22 working days per month. Catastrophic health expenditure was defined as total expenses that exceeded 20% of reported annual household income. Reduction in income was estimated from pre-illness minus the post-illness reported income for participants with non-zero pre-illness income.

### Statistical analysis

To detect overall disease prevalence of at least 5% with 1·5% precision, 90% power, and an α=0·05, the calculated sample size was 1544 participants. Descriptive statistics were used to report categorical demographics and disease prevalence. Data were reported according to distribution (mean, SD [for parametric data]; median and IQR [for non-parametric data]). Mean costs are reported in appendix 3 (p 32). Pearson's χ^2^ test was used for binary variables. Mantel–Haenszel's test for linear trend was used to assess associations between ordinal and binary variables. Odds ratios (ORs) were reported for analyses of binary outcomes. Follow-up time was calculated from hospital admission until the date of death or censoring (ie, the date of loss to follow-up or the end of follow-up), except for HRQoL for which final observations were included regardless of date (no censoring). Data on all-cause mortality were summarised using Kaplan–Meier plots. Hazard ratios (HRs) were calculated using multivariable Cox regression analyses with adjustment for age, sex, site, and severity of illness (using universal vital assessment [UVA] scores).^20^ Confounder-adjusted survival curves were derived using the fitted Cox model with bootstrapped CIs (n=500; using the adjustedsurv package in R). HRQoL utility and cost data were analysed using Kruskall–Wallis and Mann–Whitney *U* tests. Adjusted analyses were modelled using generalised linear models with gamma distribution and log-link function (HRQoL utility data transposed to the positive real line to enable modelling). For baseline HRQoL utility analyses, we adjusted for age, sex, site, and UVA. For analyses of final HRQoL observations, we also adjusted for the number of days since admission; we did not adjust for UVA, since UVA represents acute illness severity at the point of admission only. Cost analyses were performed separately for each country to reflect differences in health financing (free at the point of care in Malawi, but not Tanzania), but were not adjusted for site. We present results from complete case analyses in: survival data (91% completeness); HRQoL health utility analyses (78% completeness; in line with guidance);^21^ and cost data (100% completeness; appendix 3 p 31). For survival data, we conducted a sensitivity analysis for best-case and worst-case survival at 90 days for participants lost to follow-up (appendix 3 p 28). For HRQoL data, we conducted a sensitivity analysis using multiple imputation with chained equations (appendix 3 p 26). Statistical analyses were performed using Stata (MP 18.0) and figures were produced using R (4.4.1).

### Role of the funding source

The funders of the study had no role in study design, data collection, data analysis, data interpretation, or writing of the report.

## Results

**Figure 1:**
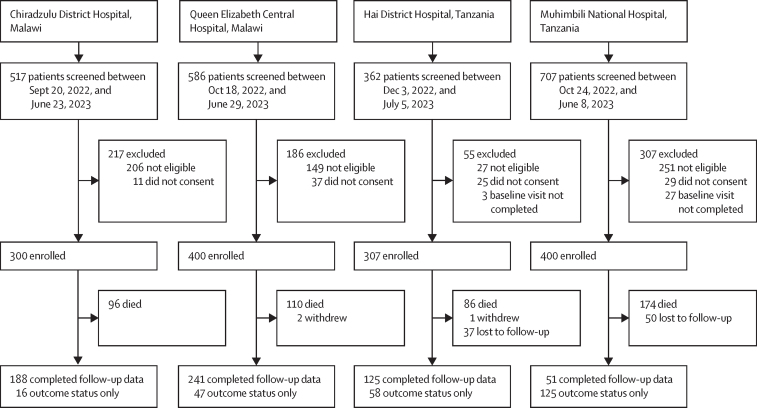
Study profile

Between Sept 20, 2022 and July 5, 2023, we recruited 1407 adults: 657 (46·7%) were female and 750 (53·3%) were male; mean age was 52·3 years (SD 18·4). Per our study protocol,^11^ 463 (32·9%) of 1407 participants were randomly assigned for cost and income data collection. Follow-up information is summarised in figure 1. Participant demographics, diagnoses, and outcomes are summarised in table 1. Results disaggregated by site show a higher burden of disease in the Muhimbili National Hospital site as a tertiary care hospital (forest plots of prevalence data provided in appendix 3 pp 17–21). Day 90 outcome status was available for 1317 (93·6%) participants and death within 90 days occurred in 446 (33·9%) participants. A description of participants lost to follow-up is available in appendix 3 (p 30). Across all four sites, systematic screening confirmed previously unidentified diagnoses of HIV in 24 (6·5%) of 368 patients living with HIV, hypertension in 185 (22·4%) of 827, diabetes in 216 (39·5%) of 547, and chronic kidney disease in 117 (34·4%) of 340 (table 1).

**Table 1:** Participant characteristics

		Chiradzulu District Hospital, Malawi*(n=300)	Queen Elizabeth Central Hospital, Malawi*(n=400)	Hai District Hospital, Tanzania*(n=307)	**Muhimbili National Hospital, Tanzania**†**(n=400)**
Mean age (SD), years		47·4 (17·6)	45·2 (17·0)	59·7 (21·2)	57·4 (13·9)
Sex					
	Female	150 (50·0%)	142 (35·5%)	192 (62·5%)	173 (43·3%)
	Male	150 (50·0%)	258 (64·5%)	115 (37·5%)	227 (56·8%)
Clinical frailty^19^					
	Not frail (clinical frailty scale <5)	190 (63·3%)	193 (48·3%)	222 (72·3%)	56 (14·0%)
	Frail (clinical frailty scale 5–6)	59 (19·7%)	161 (40·3%)	43 (14·0%)	105 (26·3%)
	Severely frail (clinical frailty scale ≥7)	51 (17·0%)	46 (11·5%)	42 (13·7%)	239 (59·8%)
Disability (Washington Short Group Set on Functioning)‡					
	With disability	93 (31·0%)	184 (46·0%)	137 (44·6%)	87 (21·8%)
	Without disability	207 (69·0%)	216 (54·0%)	170 (55·4%)	313 (78·3%)
Universal vital assessment					
	Low risk (universal vital assessment 0–1)	118 (39·3%)	172 (43·0%)	187 (60·9%)	184 (46·0%)
	Medium risk (universal vital assessment 2–4)	151 (50·3%)	183 (45·8%)	88 (28·7%)	158 (39·5%)
	High risk (universal vital assessment >4)	31 (10·3%)	45 (11·3%)	32 (10·4%)	58 (14·5%)
Baseline health-related quality-of-life utility score§					
Median health utility score (IQR)		0·53 (0·13 to 0·70)	0·58 (0·23 to 0·70)	0·31 (−0·17 to 0·74)	0·42 (0·07 to 0·64)
Mean EQ-5D-5L visual analogue score (SD)		55·2 (17·7)	62·9 (13·6)	57·7 (17·5)	61·4 (16·4)
Mean mid upper arm circumference (SD), cm		25·3 (32·5)	26·4 (3·2)	26·2 (5·1)	25·9 (3·6)
Median estimated glomerular filtration rate (IQR), mL/min per 1·73 m^2^		96·0 (56·9 to 118·0)	100·7 (63·3 to 120·4)	82·0 (51·1 to 108·9)	11·7 (5·0 to 43·7)
Mean haemoglobin (SD), g/L¶		128·1 (38·4)	137·7 (38·8)	122·2 (28·7)	109·2 (36·2)
HIV infection‖					
	Known infection	111 (37·0%)	130 (32·5%)	45 (14·7%)	58 (14·5%)
	Newly diagnosed	6 (2·0%)	10 (2·5%)	5 (1·6%)	3 (0·8%)
	Uninfected	181 (60·3%)	254 (63·5%)	256 (83·4%)	339 (84·8%)
	Unknown status	2 (0·7%)	6 (1·5%)	1 (0·3%)	0
Hypertension‖**					
	Known diagnosis	69 (23·0%)	103 (25·8%)	108 (35·2%)	362 (90·5%)
	Newly diagnosed	48 (16·0%)	72 (18·0%)	42 (13·7%)	23 (5·8%)
	None or absent	183 (61·0%)	225 (56·3%)	157 (51·1%)	15 (3·8%)
Diabetes‖					
	Known diagnosis	15 (5·0%)	39 (9·8%)	50 (16·3%)	227 (56·8%)
	Newly diagnosed	45 (15·0%)	62 (15·5%)	64 (20·8%)	45 (11·3%)
	None or absent	240 (80·0%)	299 (74·8%)	193 (62·9%)	128 (32·0%)
Chronic kidney disease‖					
	Known diagnosis	1 (0·3%)	2 (0·5%)	6 (2·0%)	214 (53·5%)
	Newly diagnosed	7 (2·3%)	31 (7·8%)	14 (4·6%)	65 (16·3%)
	None or absent	292 (97·3%)	367 (91·8%)	287 (93·5%)	121 (30·3%)
Depression‖					
	Present	11 (3·7%)	38 (9·5%)	56 (18·2%)	61 (15·3%)
	None or absent	289 (96·3%)	362 (90·5%)	251 (81·8%)	339 (84·8%)
Heart failure††					
	Known diagnosis	3 (1·0%)	13 (3·3%)	16 (5·2%)	16 (4·0%)
	Newly diagnosed	31 (10·3%)	41 (10·3%)	11 (3·6%)	58 (14·5%)
	None or absent	266 (88·7%)	341 (85·3%)	265 (86·3%)	321 (80·3%)
	Unknown	0	5 (1·3%)	15 (4·9%)	5 (1·3%)
Cerebrovascular accident††					
	Known diagnosis	14 (4·7%)	19 (4·8%)	21 (6·8%)	30 (7·5%)
	Newly diagnosed	4 (1·3%)	17 (4·3%)	19 (6·2%)	17 (4·3%)
	None or absent	282 (94·0%)	359 (89·8%)	253 (82·4%)	348 (87·0%)
	Unknown	0	5 (1·3%)	14 (4·6%)	5 (1·3%)
Chronic obstructive pulmonary disease††					
	Known diagnosis	1 (0·3%)	3 (0·8%)	6 (2·0%)	2 (0·5%)
	Newly diagnosed	0	9 (2·3%)	4 (1·3%)	2 (0·5%)
	None or absent	299 (99·7%)	383 (95·8%)	283 (92·2%)	391 (97·8%)
	Unknown	0	5 (1·3%)	14 (4·6%)	5 (1·3%)
Ischaemic heart disease††					
	Known diagnosis	1 (0·3%)	0	0	1 (0·3%)
	Newly diagnosed	0	2 (0·5%)	1 (0·3%)	1 (0·3%)
	None or absent	299 (99·7%)	393 (98·3%)	291 (94·8%)	393 (98·3%)
	Unknown	0	5 (1·3%)	15 (4·9%)	5 (1·3%)
Chronic liver disease††					
	Known diagnosis	6 (2·0%)	0	9 (2·9%)	0
	Newly diagnosed	6 (2·0%)	11 (2·8%)	6 (2·0%)	3 (0·8%)
	None or absent	288 (96·0%)	384 (96·0%)	277 (90·2%)	392 (98·0%)
	Unknown	0	5 (1·3%)	15 (4·9%)	5 (1·3%)
Current tobacco smoker		22 (7·3%)	51 (12·8%)	11 (3·6%)	6 (1·5%)
Current alcohol use		45/298 (15·1%)	91/397 (22·9%)	59/400 (19·2%)	45/398 (11·3%)
Number of long-term conditions					
	0	78 (26·0%)	102 (25·5%)	62 (20·2%)	0
	1	100 (33·3%)	101 (25·3%)	91 (29·6%)	8 (2·0%)
	≥2	122 (40·7%)	197 (49·3%)	154 (50·2%)	392 (98·0%)
Inpatient mortality		26 (8·7%)	70/399 (17·5%)	18/271 (6·6%)	73/342 (21·3%)
30-day mortality		36 (12·0%)	86/399 (21·6%)	59/281 (21·0%)	141/386 (36·5%)
90-day mortality		80 (26·7%)	110/398 (27·6%)	82/269 (30·5%)	174/350 (49·7%)
90-day readmission‡‡		27/201 (13·4%)	33/285 (11·6%)	20/146 (13·7%)	78/180 (43·3%)
90-day mortality or readmission		107/281 (38·1%)	143/395 (36·2%)	102/228 (44·7%)	252/354 (71·2%)

Data are n (%) or n/N (%), unless otherwise stated. Percentages might not add to 100 due to rounding.

* Receives referrals from primary care facilities.

† Does not routinely receive referrals from primary care facilities.

‡ Disability was determined, according to the Washington Group-Short Set on Functioning,^20^ as anyone having at least a lot of difficulty on at least one of the five questions (ie, on vision, mobility, self-care, cognition, or communication).

§ Health-related quality of life utility score, based on EQ-5D-5L responses, where 1 is perfect health, 0 reflects a state equivalent to death and <0 is a state considered to be worse than death.^21^ Universal vital assessment is based on validated threshold levels for low, medium, and high risk.^22^

¶ Haemoglobin concentrations adjusted for altitude >1000 m per WHO guidance^23^ (−2 g/L for participants in Blantyre, Chiradzulu, and Hai Districts), and by smoking status (half a packet to 1 packet per day −3 g/L; 1–2 packets per day −5g/L; ≥2 packets per day −7 g/l).

‖ Primary outcome conditions.

** Hypertension case definition includes participants with single disease and is inclusive of a lower blood pressure threshold for participants with cardiovascular risk factors.

†† Secondary outcome conditions (diagnoses captured from patient records).

‡‡ 90-day readmission data do not include data for participants who died before day 90.

**Figure 2:**
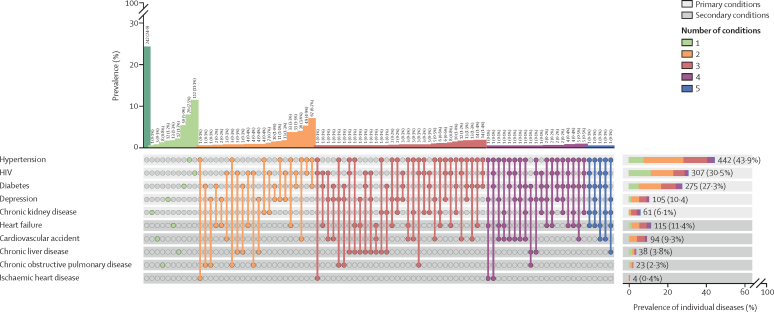
Prevalence and characteristics of multimorbidity among people admitted to hospital in Malawi and Tanzania This figure summarises disease prevalence across two hospitals in Malawi (ie, the Queen Elizabeth Central Hospital and the Chiradzulu District Hospital) and one in Tanzania (ie, the Hai District Hospital). The x-axis shows the conditions included. Numbers represent the count (n) and percentage (%). When single circles are shown, the corresponding vertical barchart shows the prevalence of the single condition. When there are two or more circles, the corresponding vertical barchart shows the prevalence of coexisting conditions. For example, above the green filled circle for HIV alone, the vertical bar shows the prevalence of participants with HIV alone (112 [11·1%] participants); above the orange filled circles for both HIV and diabetes, the vertical barchart shows the prevalence of participants with coexistent HIV and diabetes but no additional conditions (33 [3·3%] participants). The horizontal barchart shows the prevalence of participants with each individual condition, stratified by colour to depict the number of additional coexistent conditions. Primary conditions diagnosed through study procedures (ie, HIV, hypertension, diabetes, chronic kidney disease, and depression) are highlighted light grey; secondary conditions captured from clinical records (ie, heart failure, cerebrovascular accident, chronic liver disease, chronic obstructive pulmonary disease, and ischaemic heart disease) are highlighted in darker grey. The dark green bar represents those with no long-term conditions.

We identified multimorbidity in 473 (47·0% [95% CI 43·9–50·5]) of 1007 participants; 292 (29·0% [26·2–32·0]) had one long-term condition and 242 (24·0% [21·4–26·7%) had no long-term conditions. We found 294 (29·2%) participants with two conditions, 137 (13·6%) with three conditions, 37 (3·7%) with four conditions, and five (0·5%) with five conditions. Figure 2 shows the prevalence of individual diseases, and disease constituents among participants with multimorbidity (site-specific results available in appendix 3 pp 13–16). We detected a statistically higher prevalence of multimorbidity in females (248 [51·2%] of 484) compared with males (225 [43·0%] of 523); OR 1·39 (95% CI 1·08–1·80). The most common long-term conditions were hypertension (442 [43·9%; 95% CI 40·8–47·0] of 1007), HIV (307 [30·5%; [27·6–33·3]), and diabetes (275 [27·3%; 24·6–30·1]). Among participants with creatinine measurements on admission, we identified renal dysfunction (estimated glomerular filtration rate <60 mL/min per 1·73 m^2^ among 138 (13·9% [11·7–16·0%]) of 994. Chronic kidney disease was identified in 37 (4·4% [3·1–6·0%]) of 839 participants based upon estimated glomerular filtration rates measured at day 90 follow-up. However, chronic kidney disease prevalence estimates increased to 6·1% (4·6–7·5) (61 of 1007) when we extended our diagnostic criteria to include clinical records and medical history (appendix 3 p 11). We observed high levels of frailty (402 [39·9%] of 1007) and disability (414 [41·1%]) among participants (table 1). Participants with multimorbidity were more unwell at presentation (higher UVA; p<0·0001); more frail (p<0·0001); and more likely to have disability (p<0·0001) compared to participants with single and no long-term conditions (appendix 3 pp 24–25).

We detected multimorbidity in 195 (63·5% [95% CI 58·1–68·9]) of 307 participants living with HIV, 225 (81·8% [77·3–86·4]) of 275 participants with diabetes, 366 (82·8% [79·3–86·3]) of 442 participants with hypertension, and 60 (98·4% [95·2–100·0]) of 61 participants with chronic kidney disease. The most common end-organ conditions were heart failure (115 [11·4%; 9·4–13·4]) of 1007); cerebrovascular accident (94 [9·3%; 7·5–11·1]); and chronic liver disease (38 [3·8%; 2·6–5·0]; figure 2).

Information on disease control is given in table 2. Among participants with hypertension, 530 (64·1%) of 827 participants had poorly controlled blood pressure (grade 1 hypertension or higher). Among participants with diabetes, 316 (61·0%) of 518 had poorly controlled diabetes (glycated haemoglobin [HbA_1c_] ≥7·0% [≥53 mmol/mol]) and 36 (6·9%) of 518 were hypoglycaemic on admission (ie, blood sugar <4 mmol/L). Summarised HbA_1c_ data at baseline and at day 90 (for survivors) are available in appendix 3 (p 28). For those with HIV, 198 (65·8%) of 301 had adequately controlled HIV (viral load <200 copies per ml), 79 (26·2%) had poorly controlled HIV (viral load ≥200 copies per ml), and 24 (8·0%) were newly diagnosed. Of the participants with established diagnoses, 174 (49·3%) of 353 with diabetes, 304 (47·4%) of 642 with hypertension, and 312 (90·7%) of 344 with HIV were taking disease-specific medications. We found no evidence of a linear association between disease control status among participants with or without multimorbidity for hypertension control, diabetes control, or HIV control (table 2).

**Table 2:** Disease control, by disease and multimorbidity

		**Total**	**Single long-term condition**	**≥2 long-term conditions**	**Mantel-Haenszel test for linear trend, odds ratio (95% CI); p value**
Blood pressure control		..	..	..	..
	Data available	**873**	**102**	**771**	..
	Hypotension*† (systolic <90 mm Hg)	67 (7·7%)	23 (22·5%)	44 (5·7%)	..
	Hypertension with good blood pressure control (<140/90 mm Hg)	276 (31·6%)	18 (17·6%)	258 (33·5%)	0·87 (0·69–1·07); p=0·18
	Grade 1 hypertension (≥140/90 mm Hg)	236 (27·0%)	29 (28·4%)	207 (26·8%)	..
	Grade 2 hypertension (≥160/100 mm Hg)	169 (19·4%)	20 (19·6%)	149 (19·3%)	..
	Hypertensive crisis (≥180/120 mm Hg)	125 (14·3%)	12 (11·8%)	113 (14·7%)	..
Diabetes control		..	..	..	..
	Data available	518	48	470	
	Hypoglycaemia* (<4 mmol/L)‡	36 (6·9%)	2 (4·2%)	34 (7·2%)	..
	Very good control (<6·5% [<48 mmol/mol])	73 (14·1%)	2 (4·2%)	71 (15·1%)	0·90 (0·68–1·19); p=0·46
	Good control (6·5–6·9% [48–52 mmol/mol])	93 (18·0%)	16 (33·3%)	77 (16·4%)	..
	Poor control (7·0–7·9% [53–64 mmol/mol])	124 (23·9%)	7 (14·6%)	117 (24·9%)	..
	Very poor control (>8·0% [>64 mmol/mol])	192 (37·1%)	21 (43·8%)	171 (36·4%)	..
HIV control		..	..	..	..
	Data available	301	98	203	..
	New HIV diagnosis*	24 (8·0%)	8 (8·2%)	16 (7·9%)	..
	Undetectable viral load (<50 copies per ml)	177 (58·8%)	56 (57·1%)	121 (59·6%)	0·99 (0·85–1·15); p=0·86
	Controlled (50–199 copies per ml)	21 (7·0%)	8 (8·2%)	13 (6·4%)	..
	Uncontrolled (200–999 copies per ml)	15 (5·0%)	5 (5·1%)	10 (4·9%)	..
	Very poor control (1000–9999 copies per ml)	6 (2·0%)	2 (2·0%)	4 (2·0%)	..
	High infectivity (≥10 000 copies per ml)	58 (19·3%)	19 (19·4%)	39 (19·2%)	..

* Hypotension and hypoglycaemia superseded other measures of control as acute presentations. Hypotension, hypoglycaemia, and new HIV diagnoses were not included in the tests for linear trend of odds. Codes used for each category included were equally spaced.

† Participants with hypotension but without a previous diagnosis of hypertension were included to present the full range of blood pressure control in this cohort.

‡ Random blood glucose level on admission.

**Figure 3:**
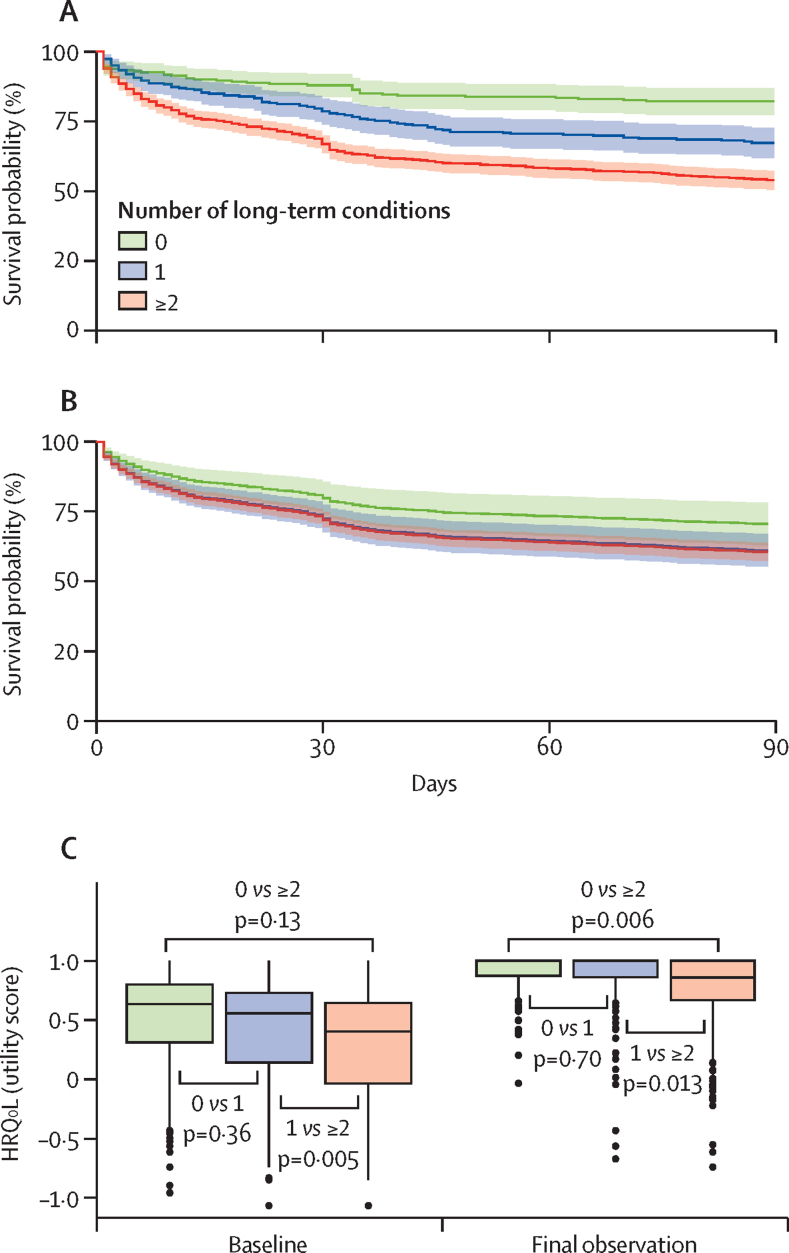
Kaplan–Meier survival plots, by number of long-term conditions (A) Unadjusted Kaplan–Meier plot. (B) Confounder-adjusted survival curves, based on the Cox regression model with adjustments for age, sex, universal vital assessment score, and site. (C) Box and whisker plot of HRQoL utility scores at baseline, and among survivors at the final observation. p values reflect multivariable generalised linear model analyses (gamma distribution), adjusted by age, sex, site, and universal vital assessment (baseline); and age, sex, site, and number of days in study (final observation). No long-term conditions, n=242; single long-term condition, n=300; and ≥2 long-term conditions, n=865. HRQoL=health-related quality of life.

Unadjusted analyses detected lower HRQoL utility scores among participants with two or more long-term conditions compared with those with no long-term conditions at both baseline (p<0·0001) and final observation (p<0·0001; appendix 3 p 26). However, after adjustment there was no statistical difference in HRQoL between participants with two or more long-term conditions and those with no long-term conditions at baseline (p=0·13; figure 3; appendix 3 p 26). At the final observation, adjusted analyses detected significantly lower HRQoL in patients with two or more conditions compared with those with one (p=0·013) and those with no long-term conditions (p=0·006), respectively (appendix 3 p 26). Findings were consistent using multiple imputation with chained equations for missing values (appendix 3 p 26).

Demographic details of the 463 participants randomly assigned for the health economic data collection are provided in appendix 3 (p 31). Regardless of multimorbidity status, total costs were lower in Malawi than Tanzania, with non-medical costs being the main cost driver in Malawi, and medical costs in Tanzania (table 3; appendix 3 p 33). Multivariable generalised linear model analyses revealed that participants from Malawi with multimorbidity faced higher non-medical costs than those with a single condition (relative effect [RE] 1·6 [95% CI 1·1–2·4]; p=0·023), higher indirect costs (16·0 [1·2–218·3]; p=0·038) but had higher post-hospitalisation income (2·6 [1·3–5·4]; p=0·01) compared with those with no long-term conditions. In Tanzania, multimorbid participants had higher medical costs (RE 2·8 [95% CI 1·7–4·6]; p <0·0001), indirect costs (22·7 [7·0–73·3]; p<0·0001), pre-hospitalisation (5·8 [3·0–11·2]; p<0·0001) and post-hospitalisation (5·3 [2·8–10·2]; p<0·0001) personal income, and household income (4·80 [2·6–8·9]; p <0·0001) than those with one long-term condition (appendix 3 p 33).

**Table 3:** Participant costs, income effects, and catastrophic costs, Malawi and Tanzania

		**Total**	**Number of long-term conditions**			p value*
			0	1	≥2	
**Malawi**						
**Participants**		**233**	**52**	**79**	**102**	**..**
Participant costs, US$						
	Medical costs†	0·00 (0·00–0·00)	0·00 (0·00–0·00)	0·00 (0·00–0·00)	0·00 (0·00–0·00)	0·939
	Non-medical costs‡	10·71 (5·64–19·92)	10·24 (4·04–21·71)	10·34 (6·11–15·98)	11·47 (5·83–22·55)	0·348
	Indirect costs§	0·00 (0·00–0·00)	0·00 (0·00–3·20)	0·00 (0·00–0·00)	0·00 (0·00–0·00)	0·599
	Total costs	14·85 (7·52–33·83)	14·71 (7·19–25·84)	12·22 (7·80–33·83)	18·80 (7·24–45·11)	..
Personal monthly income						
	Pre-hospitalisation	32·89 (9·40–65·79)	37·59 (5·64–58·74)	28·19 (9·40–56·39)	34·30 (9·40–93·04)	0·674
	Post-hospitalisation	14·10 (0·00–46·99)	0·02 (0·00–46·99)	18·80 (0·00–46·99)	10·57 (0·00–46·99)	0·542
	Individual income loss¶	0·00 (0·00–23·49)	0·00 (0·00–28·19)	0·00 (0·00–14·10)	0·01 (0·00–28·19)	0·448
	Household annual income	338·32 (84·58–845·81)	563·87 (0·56–845·81)	338·32 (112·77–733·03)	338·32 (45·11–902·20)	0·759
	Catastrophic costs‖	65/232 (28·0%)	13/51 (25·5%)	18/79 (22·8%)	34/102 (33·3%)	0·264
**Tanzania**						
**Participants**		**230**	**18**	**33**	**179**	**..**
Participant costs, US$						
	Medical costs†	70·53 (30·93–197·27)	35·37 (22·48–57·33)	38·77 (22·68–60·83)	104·55 (41·04–265·61)	0·0001
	Non-medical costs‡	5·77 (0·00–15·67)	7·84 (2·89–18·56)	10·31 (3·71–21·03)	4·54 (0·00–14·44)	0·008
	Indirect costs§	0·00 (0·00–26·25)	0·00 (0·00–16·87)	0·00 (0·00–0·00)	0·00 (0·00–67·49)	0·119
	Total costs	106·16 (53·00–309·51)	58·55 (38·30–89·50)	56·61 (39·01–79·81)	148·06 (61·86–392·07)	..
Personal monthly income						
	Pre-hospitalisation	41·24 (0·00–123·73)	16·50 (0·00–61·86)	0·00 (0·00–41·24)	61·86 (0·00–164·97)	0·0001
	Post-hospitalisation	24·75 (0·00–103·11)	3·09 (0·00–49·49)	0·00 (0·00–32·99)	41·24 (0·00–144·35)	0·002
	Individual income loss**	20·62 (0·00–41·24)	0·82 (0·00–32·99)	0·00 (0·00–24·75)	20·62 (0·00–41·24)	0·582
	Household annual income	816·62 (0·00–2474·59)	37·12 (0·00–643·39)	0·00 (0·00–742·38)	1088·82 (0·00–3216·97)	0·0001
	Catastrophic cost‖	115/230 (50·0%)	10/18 (55·6%)	23/33 (69·7%)	82/179 (45·8%)	0·037

Data are n/N (%) or median (IQR), unless otherwise specified. Cost and income data were collected in national currencies (ie, Tanzanian shillings and Malawian Kwacha then converted to US$ using exchange rates for June 30, 2023, of 2424·64 for Tanzania and 1064·07 for Malawi as found on Oanda.

* p values were generated using the Kruskal-Wallis test.

† Medical costs comprise medications, tests, and medical supplies.

‡ Non-medical costs comprise transport and food for participants and their guardians.

§ Indirect costs are estimated for each participant based on the reported number of workdays lost multiplied by the reported mean monthly pre-illness income, divided by 22 days. A high proportion of participants had no income, reflected within the reported values.

¶ Individual income loss was calculated when participants had income of >0 at baseline: n=195: no long-term conditions (n=42); one long-term condition (n=67); and two long-term conditions (n=86).

‖ Participants for whom total costs were >20% of their annual reported household income (we collected monthly household income and multiplied by 12 months to estimate annual).

** Individual income loss calculated when participants had income of >0 at baseline: n=158: no long-term conditions (n=11); one long-term conditions (n=15); and two long-term conditions (n=132).

Across all four sites, the 90-day all-cause mortality was 31 (13·5%) of 230 participants with no long-term conditions, 80 (28·3%) of 283 participants with one long-term condition, and 335 (41·7%) of 804 among participants with multimorbidity (appendix 3 p 27). Our unadjusted Cox regression model showed an increased risk of mortality for participants with one long-term condition (HR 2·0 [95% CI 1·4–2·8]), and participants with two or more long-term conditions (3·1 [2·2–4·2]) compared with participants with no long-term conditions (appendix 3 p 27). We found an increased risk of mortality for participants with two or more conditions compared with one long-term condition (1·6 [1·3–2·0]). When we adjusted for age, sex, site, and UVA score, this significant increase in mortality risk persisted although HRs were smaller both for participants with one long-term condition (1·5 [1·0–2·1]), and for participants with two or more long-term conditions (1·5 [1·1–2·1]). Participants with two or more long-term conditions did not have increased risk of mortality compared with one long-term condition (HR 1·0 [CI 0·8-1·3]) after adjustment (appendix 3 p 27). Sensitivity analyses for best-case and worst-case scenarios for participants lost to follow-up were consistent with our complete case analysis (appendix 3 p 28). Kaplan–Meier survival plots are shown in figure 3 (appendix 3 p 29).

## Discussion

In this prospective multicentre cohort study among participants who were acutely ill and hospitalised in Malawi and Tanzania, multimorbidity was common (47·0%) and associated with high 90-day mortality (41·7%). We found 43·9% prevalence of hypertension, 30·5% of HIV, and 27·3% of diabetes. We observed poor hypertension and diabetes disease control, increasing the risk of end-organ diseases such as heart failure, stroke, and chronic kidney disease. We observed high viral suppression for HIV, showing that successful delivery of protocolised health care in this setting is possible. Participants with long-term conditions suffered higher rates of 90-day mortality than participants with no long-term conditions after adjustment for age, sex, and physiological dysfunction. We recommend interventional studies to inform how health systems should address rising rates of multimorbidity in sub-Saharan Africa.

The prevalence of heart failure was 11·4% and was 9·3% for cerebrovascular events from medical records (clinical diagnostic criteria), consistent with a systematic review of chronic disease prevalence estimates from hospitals across sub-Saharan Africa.^2^ Poor disease control was identified in 64·1% of participants with hypertension, 61·0% with diabetes, and 26·2% with HIV, with less than half of participants with known hypertension and diabetes taking disease-specific medication at hospital admission. In contrast, 90·7% of participants with known HIV were taking antiretroviral medications at admission. Similarly, our study procedures identified high rates of newly diagnosed NCDs (particularly hypertension, diabetes, and heart failure), and low rates of newly diagnosed HIV. This finding likely reflects constrained diagnostic availability for NCDs, compared with well established vertical programmes for HIV diagnosis. Under-diagnosis and poor disease control, particularly for NCDs, is a challenge recognised in both hospital and community settings of sub-Saharan Africa.^2^, ^22^, ^24^

Contextual factors impact on patterns and management of multimorbidity in sub-Saharan Africa compared with high-income countries.^25^ For example, the success of antiretroviral drug roll-out for HIV infection in sub-Saharan Africa means people are living with this chronic communicable disease for much longer.^26^ Both HIV infection and the antiretroviral drugs used for management can drive the onset and progression of NCDs, but these diseases are frequently not addressed by existing vertically orientated health systems.^7^ We found 39·6% of participants with multimorbidity were aged 60 years or younger in our cohort, a finding aligned with previous research showing earlier onset of both type 2 diabetes and hypertension in African populations.^27^ Earlier life presence of multimorbidity has been noted in African populations compared with high-income countries.^28^

As anticipated, our hospital-based estimates of multimorbidity are higher than estimates from community settings in sub-Saharan Africa (28·2% [95% CI 15·6–40·8]).^29^ In addition to different patient populations, the limited availability of essential diagnostics could partially explain these differences as the current study in hospital settings identified conditions.^29^ Participants with multimorbidity suffered higher medical and indirect costs compared with those without multimorbidity in Tanzania, with a less pronounced effect in Malawi. Higher personal and household income in people with multimorbidity in Tanzania could explain the lower incidence of catastrophic costs in this group, relative to those with fewer long-term conditions.

Our study findings reflect the contextual differences in health financing between free at the point of use health care in Malawi, and the reformed user fee system in Tanzania. As health financing reforms throughout sub-Saharan Africa coincide with the rise of NCDs, people with multimorbidity are particularly vulnerable to delayed and reduced health-care access; increased economic costs; and poor health outcomes. At the macroeconomic level this combination of costs and reduced health, particularly among working age people commonly affected by multimorbidity can impair growth and economic development. We found low health utility (ie, numerical HRQoL scores) at baseline in our cohort, in alignment with high levels of disability, frailty, and severe acute illness from clinical data. We observed high mortality and poor health utility among survivors with multimorbidity, indicative that preventable secondary complications are not being sufficiently addressed. Early diagnosis and management are required to prevent unplanned hospital admissions with high associated costs for patients and the health system.

To the best of our knowledge, this study is the first detailed description of multimorbidity in hospital settings in sub-Saharan Africa.^2^ We used commercially available Conformité Européenne-marked point-of-care tests as a pragmatic diagnostic approach for our study setting in which laboratory services are constrained. This ensured a standardised diagnostic approach across all four sites to systematically screen consecutively enrolled participants for multimorbidity, following internationally recognised diagnostic reference criteria for our defined set of primary conditions.^11^ We were able to deliver the study despite intermittent recruitment pauses in Malawi due to a national cholera outbreak and due to Tropical Cyclone Freddy. We faced logistical challenges around delayed delivery of point-of-care cartridges and device malfunctions (particularly affecting the iSTAT device [Abbott Park, IL, USA]) during periods of extreme heat across all sites. However, there are several limitations to our study. First, our multimorbidity estimates are based on acute medical admissions so might not be generalisable across the whole hospital population. A European cohort study identified high rates of multimorbidity in elective and emergency surgical patients.^30^ Second, we were not able to differentiate between type 1 and type 2 diabetes. Third, diagnosis of chronic kidney disease requires evidence of persistent abnormality for 90 days,^14^ meaning our estimates are prone to survivorship bias. Unavailable modalities of the Kidney Disease: Improving Global Outcomes guidelines^21^ (such as albumin:creatinine ratio estimation) were excluded, which could result in an underestimation of chronic kidney disease in our cohort.^14^ Fourth, we were unable to use Muhimbili National Hospital as a site to estimate multimorbidity prevalence due to the implementation of new referral structures immediately before study commencement. Fifth, our diagnostic certainty is lower for conditions captured through clinical notes (ie, secondary conditions), which reflects limited diagnostic capacity and budgetary constraints to provide more complex diagnostics (eg, CT scans for stroke). Sixth, the smaller sample size for cost and income analysis potentially reduced our ability to determine differences between participants with and without multimorbidity. Seventh, we observed heterogeneity in disease constituents of multimorbidity between countries, such that a one-size-fits-all approach to multimorbidity management is most likely not appropriate. We also observed differences in participant eligibility between sites, reflective of differential hospital admission pathways and higher loss to follow-up in Tanzania (most commonly due to migration out of the hospital catchment area) compared with Malawi.

In summary, our prospective multicentre cohort study in Malawi and Tanzania reveals a high prevalence of multimorbidity and mortality among adults who are hospitalised, with a high prevalence of hypertension, diabetes, and HIV. Poor control of hypertension and diabetes was common, in contrast with better-managed HIV. Our results indicate that multimorbidity places a major economic and health-related burden on patients in these contexts. These findings suggest a crucial need for prevention, early identification, and improved long-term disease management to reduce complications and mortality. Health systems approaches are required to address health-care worker training to better recognise and manage multimorbidity; sustainable financing to address diagnostic and medication gaps; and improved linkage between primary and secondary care to improve disease control and reduce the risk of acute decompensated hospital presentations.

### Multilink Consortium

### Contributors

### Equitable partnership declaration

### Data sharing

An anonymised study dataset can be shared within Malawi and Tanzania in line with local data sharing policies. Requests for data sharing can be presented to the MultiLink management committee via our programme manager, Amy Smith (amy.smith@lstmed.ac.uk).

## Declaration of interests

PD has received funding from the UK National Institute for Health and Care Research, the Medical Research Council, Innovate UK, and Wellcome Trust to conduct related clinical research in the UK; and was Deputy Medical Director of The National Institute for Health and Care Research, UK (2022–24). MPR has received additional funding from the US National Institute of Allergy & Infectious Diseases to conduct sepsis research in Tanzania; is the Chairperson on the Data Safety Monitoring Board for A Randomized Clinical Trial of Early Empiric Anti-Mycobacterium Tuberculosis Therapy for Sepsis in sub-Saharan Africa (ATLAS); and MPR's research programme includes a member that has a research collaboration with Cepheid that includes receipt of materials and payment of research-associated expenses. MN is a member of the Primary Trauma Care Global Advisory Group, the African Federation of Emergency Medicine Board, and the Medical Council of Malawi Board. All other authors declare no competing interests.

## Supplementary Materials

Chichewa translation of the abstract

Kiswahili translation of the abstract

Supplementary appendix 3

Equitable Partnership Declaration
