# Supplementary material for: The burden of multimorbidity-associated acute hospital admissions in Malawi and Tanzania: a prospective multicentre cohort study
Source: Lancet Glob Health. 2025 Jun 25;13(7):e1279–90. doi: 10.1016/S2214-109X(25)00113-5 (PMC12208785; doi:10.1016/S2214-109X(25)00113-5)
Supplement: Chichewa translation of the abstract [file mmc1.pdf]

THE LANCET
Global Health

## Supplementary appendix 1

This translation in Chichewa was submitted by the authors and we reproduce it as supplied. It has not been peer reviewed. *The Lancet*'s editorial processes have only been applied to the original in English, which should serve as reference for this manuscript.

Kutanthauzila kwa mu Chichewa uku kwachokela kwa alembi a nkhaniyi ndipo tayipeleka kwa inu m'mene tinayilandilila. Mawu a mu Chichewa sanawunikilidwenso kapena kukonzedwa. Nkhani ya mu Chingelezi yokha ndi imene yadutsa mu ukonzi wa Lancet, kotelo kuti nkhani ya mu Chingeleziyi ndi imene ikuyimilila mokwanila nkhani yonse imene yalembedwa.

Supplement to: Spencer SA, Yongolo NM, Simiyu IG, et al. The burden of multimorbidity-associated acute hospital admissions in Malawi and Tanzania: a prospective multicentre cohort study. *Lancet Glob Health* 2025; **13:** e1279–90.

**Mavuto okhudza chisamaliro cha anthu amene agonetsedwa muchipatala chifukwa chokhudzidwa ndi matenda angapo a m'gonagona ku maiko a Malawi ndi Tanzania: kafukufuku wolondoloza magulu osiyanasiyana a anthu odwala mu zipatala.**

## Kufotokoza mwachidule

### Mawu oyamba

Pa dziko lonse lapansi, mavuto amene amabwera chifukwa chakuti anthu apezeka kapena kukhudzidwa ndi matenda angapo a m'gonagona - kukhala ndi matenda awiri kapena kuposera apo kwa nthawi yaitali (m'gonagona) - akuchulukira. Kupezeka kwa chithandizo choyambilira cha mankhwala kummwera kwa Africa ndi kochepa, ndipo izi zimatanthauza kuti nthawi zambiri, kugonekedwa mu chipatala chachikulu ndi njira yokhayo yowonetsera zizindikiro za vuto lalikulu la matenda angapo a m'gonagona. Kafukufuku wolondoloza magulu osiyanasiyanayu a anthu odwala mu zipatala zosiyanasiyana, cholinga chake chinali chofuna kufotokoza mavuto, matenda osiyanasiyana komanso zotsatira za matenda angapo a m'gonagona pakati pa odwala omwe agonekedwa mu chipatala ku maiko a Malawi ndi Tanzania.

### Njira zochitira kafukufuku

Anthu akuluakulu (a zaka zoposera 18) omwe anagonekedwa muzipatala zinayi (zipatala ziwiri zazikulu, ziwiri zapaboma) chifukwa adwalika mwadzidzidzi, analowetsedwa mukafukufukuyu mkati mwa maola 24 ataonedwa ndi achipatala, ndipo anatsatiridwa kupeza zotsatira patatha masiku 90 (miyezi itatu). Tinayerekeza kuchuluka kwa matenda oyambitsidwa ndi kachilombo ka HIV, matenda a shuga, matenda a kuthamanga kwa magazi, matenda a impyo, pogwiritsa ntchito zida zovomerezeka zoyezera odwala pamalo pomwepo pamene ali, potengera zomwe odwalayo anafotokoza ndi zomwe a chipatala anapeza (n/N, %). Zotolera zokhudza umoyo ndi chuma zinaunikidwa molingana ndi nambala yapakatikati pogwiritsa ntchito mitundu yofananira. Zonse zokhudza imfa zimene zinachitika patapita masiku 90 zidafotokozedwa mwachidule ndi njira zotchedwa Kalplan Meier ndipo zinawunikidwa pogwiritsa ntchito zitsanzo zotchedwa Cox regression.

**Zotsatira zomwe zapezeka**

Tinalowetsa mukafukufukuyu anthu akuluakulu okwana 1407 (657 [46.7%] anali akazi ndipo 750 [53.3%] anali amuna; zaka za pakati 52:3 [SD 18·4]). Kuchuluka kwa matenda angapo a m'gonagona kunawunikidwa mwachindunji mwa anthu 1007 otenga nawo mbali omwe anagonekedwa muzipatala zitatu zimene zimagonetsa anthu kuchokera kumadera a kumudzi. Matenda angapo a m'gonagona anapezeka mwa otenga nawo mbali okwanira 473 (47·0%) mwa anthu 1007, ndipo anthu 292 (29.0%) ndi omwe anali ndi nthenda imodzi ya m'gonagona. Zotsatira za odwala patatha masiku 90 zinatsimikiziridwa kwa otenga nawo mbali okwana 1317 (93·6%) mwa anthu 1407. Mwa anthu amene anamwalira pakutha kwa masiku 90, imfa zinali zokwera kwambiri mwa anthu otenga nawo mbali omwe ali ndi matenda angapo a m'gonagona (335 [41·7%] mwa anthu 804; chiwerengero cha chiopsezo [HR] 1·5 [95% CI 1·1-2.1]) komanso omwe ali ndi matenda amodzi a m'gonagona (80 [28·3%] mwa anthu 283; HR 1·5 [1·0-2·1]); poyerekeza ndi omwe alibe matenda a m'gonagona (31 [13·5%] mwa anthu 230]; 1.5 [1.1-2.1]. Kwa otenga nawo mbali , zamomwe munthu amamvera paumoyo wake wa thanzi zinali zotsika kwambiri kwa otenga nawo mbali omwe ali ndi matenda angapo a m'gonagona kufanizira ndi omwe ali ndi nthenda imodzi ya m'gonagona (nambala yapakatikati 0.402 [IQR -0.037 mpaka 0.644] kusiyanitsa ndi 0.557 [IQR 0.140 mpaka 0.730], p=0.005) pamene amalowa kafukufuku, komanso pamene amawunikidwa komaliza mukafukufukuyu (0.858 [IQR 0.667 mpaka 1.00] kusiyanitsa ndi 1.00 [IQR 0.589 mpaka 1.00] motsatirana choncho, p=0.01). Ku dziko la Tanzania, panali umboni wina wa kuchuluka kwa ndalama zogwiritsa ntchito zosamalira matenda, kwa otenga nawo mbali omwe ali ndi matenda awiri kapena angapo a m'gonagona kusiyanitsa ndi anthu amene anali ndi nthenda imodzi ya m'gonagona (kuyerekeza [RE] 5.77 (2.99 – 11.15) P<0.0001).

**Kutanthauzira kafukufuku**

Ku Malawi and ku Tanzania, matenda angapo a m'gonagona amapezeka kwambiri mwa odwala omwe amagonekedwa mu chipatala ndipo amakhudzana ndi wodwalayo kukhala ndi mwayi wochepa wokhala ndi moyo akapezeka ndi matendawa, komanso kukwera kwa ndalama zogwiritsidwa ntchito posamalira matendawa. Matenda angapo a m'gonagona amakhala chiopsezo chachikulu pa umoyo wa anthu, zinthu zomwe zikufunika kusintha njira zoperekera chithandizo cha makhwala kuti tithe kuthana ndi zosowa za anthu.
