# Supplementary material for: The burden of multimorbidity-associated acute hospital admissions in Malawi and Tanzania: a prospective multicentre cohort study
Source: Lancet Glob Health. 2025 Jun 25;13(7):e1279–90. doi: 10.1016/S2214-109X(25)00113-5 (PMC12208785; doi:10.1016/S2214-109X(25)00113-5)
Supplement: Kiswahili translation of the abstract [file mmc2.pdf]

THE LANCET
Global Health

Supplementary appendix 2

This translation in Kiswahili was submitted by the authors and we reproduce it as supplied. It has not been peer reviewed. *The Lancet*'s editorial processes have only been applied to the original in English, which should serve as reference for this manuscript.

Tafsiri hii katika Kiswahili iliwasilishwa na waandishi na tunatengeneza tena kama hutolewa. Haijapitiwa. Mchakato wa hariri wa *Lancet* umetumika tu kwa asili kwa Kiingereza, ambayo inapaswa kutumika kama kumbukumbu kwa muswada hii.

Supplement to: Spencer SA, Yongolo NM, Simiyu IG, et al. The burden of multimorbidity-associated acute hospital admissions in Malawi and Tanzania: a prospective multicentre cohort study. *Lancet Glob Health* 2025; **13:** e1279–90.

Mzigo wa kulazwa hospitalini kutokana na maradhi mengi sugu nchini Malawi na Tanzania: Utafiti wa kikundi wa vituo vingi uliofuatiliwa kwa muda

Muhtasari

Utangulizi:

Mzigo wa kimataifa ni mkubwa kwa waginjwa wenye maradhi mengi sugu—yaani kuwepo kwa hali mbili au zaidi za kiafya za muda mrefu kwa mtu mmoja—unaendelea kuongezeka. Upatikanaji mdogo wa huduma za afya ya msingi katika Afrika Kusini mwa Jangwa la Sahara husababisha kulazwa hospitalini kwa dharura kuwa ndiyo ishara ya kwanza ya maradhi haya. Utafiti huu wa kikundi uliofanywa katika vituo vingi kwa kufuatilia washiriki kwa muda, ulikusudia kueleza mzigo, magonjwa yanayochangia, na matokeo ya maradhi mengi sugu kwa wagonjwa waliolazwa kwa dharura hospitalini nchini Malawi na Tanzania.

Mbinu:

Watu wazima (yaani wenye umri wa miaka ≥18) waliolazwa hospitalini katika hospitali nne (hospitali mbili za rufaa na mbili za wilaya) kwa hali za dharura za kitabibu walichaguliwa mfululizo ndani ya saa 24 tangu walipowasili na kufuatiliwa kwa siku 90. Tulikadiria kiwango cha maambukizi ya VVU, kisukari, shinikizo la damu, na ugonjwa sugu wa figo kwa kutumia vipimo vya haraka vinavyopatikana, na tukachukua taarifa za magonjwa kwa njia ya kujieleza binafsi na kutoka katika taarifa za kitabibu (n/N, %). Takwimu za kiuchumi za afya zilifupishwa kwa wastani wa kati (median) na wigo wa robo (IQR), na kuchambuliwa kwa kutumia mifano ya mstari wa jumla. Vifo vyote ndani ya siku 90 vilifupishwa kwa kutumia michoro ya Kaplan-Meier na kuchambuliwa kwa kutumia mifano ya Cox.

Matokeo:

Watu wazima 1,407 (657 [46·7%] wanawake na 750 [53·3%] wanaume; umri wa wastani ulikuwa miaka 52·3 [SD 18·4]) walishirikishwa. Tulichunguza maradhi mengi sugu kwa washiriki 1,007 waliolazwa moja kwa moja kutoka katika jamii katika hospitali tatu. Maradhi mengi sugu yalipatikana kwa washiriki 473 (47·0%) kati ya 1,007, na 292 (29·0%) walikuwa na hali moja ya muda mrefu ya kiafya. Matokeo ya siku 90 yalijulikana kwa washiriki 1,317 (93·6%) kati ya 1,407. Vifo vilivyorekebishwa kwa siku 90 vilikuwa juu kwa washiriki wenye maradhi mengi sugu (335 [41·7%] ya 804; uwiano wa hatari [HR] 1·5 [95% CI 1·1–2·1]) na kwa wale wenye hali moja ya muda mrefu (80 [28·3%] ya 283; HR 1·5 [1·0–2·1])

ikilinganishwa na wale wasio na hali ya muda mrefu ya kiafya (31 [13·5%] ya 230; HR 1·5 [1·1–2·1]). Ubora wa maisha ulihusiana na afya (HRQoL) ulikuwa wa chini zaidi kwa washiriki wenye hali zaidi ya mbili sugu ikilinganishwa na waliokuwa na hali moja (median 0.402 [IQR -0.037 hadi 0.644] dhidi ya 0.557 [IQR 0.140 hadi 0.730], p=0.005) mwanzoni, na pia katika tathmini ya mwisho (0.858 [IQR 0.667 hadi 1.00] dhidi ya 1.00 [IQR 0.589 hadi 1.00], mtawalia, p=0.01). Nchini Tanzania, gharama za matibabu kwa wagonjwa zilikuwa juu zaidi kwa washiriki wenye hali zaidi ya mbili ikilinganishwa na wale wenye hali moja (athari ya ulinganisho [RE] 5.77 (2.99 – 11.15) P<0.0001).

Hitimisho:

Maradhi mengi sugu kwa wakati mmoja ni ya kawaida kwa wagonjwa waliolazwa hospitalini nchini Malawi na Tanzania, na yanahusishwa na kiwango cha juu cha vifo na gharama kubwa za matibabu. Maradhi mengi sugu ni tishio kubwa la afya ya umma linalohitaji mabadiliko ya msingi katika utoaji wa huduma za afya ili kukidhi mahitaji ya jamii.
