# Supplementary material for: The burden of multimorbidity-associated acute hospital admissions in Malawi and Tanzania: a prospective multicentre cohort study
Source: Lancet Glob Health. 2025 Jun 25;13(7):e1279–90. doi: 10.1016/S2214-109X(25)00113-5 (PMC12208785; doi:10.1016/S2214-109X(25)00113-5)
Supplement: Supplementary appendix 3 [file mmc3.pdf]

THE LANCET
Global Health

Supplementary appendix 3

This appendix formed part of the original submission and has been peer reviewed. We post it as supplied by the authors.

Supplement to: Spencer SA, Yongolo NM, Simiyu IG, et al. The burden of multimorbidity-associated acute hospital admissions in Malawi and Tanzania: a prospective multicentre cohort study. *Lancet Glob Health* 2025; **13:** e1279–90.

Supplemental material

Table of Contents

Supplemental material 1

STROBE Checklist 3

Site characteristics 4

Case definitions 5

Hypertension definition 5

Table S 1 Hypertension control 5

Diabetes mellitus definition 5

Table S 2 Diabetes control 5

HIV definition 5

Table S 3 HIV control 6

Chronic kidney disease (CKD) definition 6

Table S 4 Chronic kidney disease (CKD) stage 6

Depression 6

Multilink cohort study: algorithms for coding clinical diagnoses. 7

Table S 5 Hypertension. Hypertension as single disease 7

Table S 6 Hypertension. Hypertension diagnosis in presence of cardiovascular disease. 8

Table S 7 Diabetes 9

Table S 8 HIV 10

Table S 9 CKD 11

Heart failure, stroke, chronic liver disease, ischaemic heart disease, chronic obstructive pulmonary disease 12

Frailty 12

Disability 12

Table S 10 Existing evidence to underpin selection of functional tools used within the cohort study. 12

Figure S 1 Prevalence and characteristics of multimorbidity among people admitted to hospital in Queen Elizabeth Central Hospital, Malawi 13

Figure S 2 Prevalence and characteristics of multimorbidity among people admitted to hospital in Chiradzulu District Hospital, Malawi 14

Figure S 3 Prevalence and characteristics of multimorbidity among people admitted to hospital in Hai District Hospital, Tanzania 15

Figure S 4 Prevalence and characteristics of multimorbidity among people admitted to hospital in Muhimbili National Hospital, Tanzania 16

Figure S 5 Prevalence of Multimorbidity by site. 17

Figure S 6 Prevalence of Multimorbidity across three sites. 17

Figure S 7 Prevalence of conditions diagnosed through study procedures: all sites 18

Figure S 8 Prevalence of conditions diagnosed through study procedures: three sites 19

Figure S 9 Prevalence of clinically diagnosed conditions: all sites 20

Figure S 10 Prevalence of clinically diagnosed conditions: three sites 21

Table S 11 Functional and mental health measures 22

Table S 12 Association between number of long-term conditions, and: age; sex; and severity of illness (universal vital assessment). 24

Table S 13 Association between number of long-term conditions and: frailty, disability. 24

Figure S 11 Prevalence of multimorbidity, by age, sex, universal vital assessment (UVA) and clinical frailty scale (CFS). 25
Table S 14 Association between number of long-term conditions and HRQoL health utility at baseline 26
Table S 15 Association between number of long-term conditions and HRQoL health utility at the final observation 26
Table S 16 Association between 90-day mortality and: frailty, disability. 27
Table S 17 Univariable and multivariable Cox regression analyses 27
Table S 18 In-patient, D30 and D90 outcomes of patients with multimorbidity, vs single long term and no long-term conditions 28
Table S 19 Sensitivity analyses showing best- and worst-case survival outcomes at D90 comparing patients with multimorbidity, vs single long-term and no long-term conditions 28
Table S 20 HbA1c at baseline and at day 90. 28
Figure S 12 Kaplan Meier survival plots for participants with 0, 1, 2, 3 and ≥4 long-term conditions 29
Table S 21 Table comparing age between consented participants and participants who declined consent. 30
Table S 22 Table comparing demographics between patients followed up at day 90 and those lost to follow-up. 30
Table S 23 Table comparing demographics between participants randomised into cost/income sub-cohort and those not randomised. 31
Table S 24 Variable missingness in multivariable analyses: survival analyses, HRQoL, cost. 31
Table S 25 Participant cost and income effects (mean), Malawi and Tanzania (USD$) 32
Table S 26 Difference in costs between participants with 0, 1 and ≥2 long-term conditions. Results from multivariable GLM model 33
References 34

STROBE Checklist

	Item No	Recommendation	Location in manuscript
**Title and abstract**	1	(*a*) Indicate the study's design with a commonly used term in the title or the abstract	Title and abstract
		(*b*) Provide in the abstract an informative and balanced summary of what was done and what was found	Abstract
**Introduction**			
Background/rationale	2	Explain the scientific background and rationale for the investigation being reported	Introduction
Objectives	3	State specific objectives, including any prespecified hypotheses	Introduction
**Methods**			
Study design	4	Present key elements of study design early in the paper	Methods – Study design; Published protocol (doi.org/10.3310/nihropenres.13512.2)
Setting	5	Describe the setting, locations, and relevant dates, including periods of recruitment, exposure, follow-up, and data collection	Methods – Study design; Published protocol (doi.org/10.3310/nihropenres.13512.2)
Participants	6	Give the eligibility criteria, and the sources and methods of selection of participants. Describe methods of follow-up	Methods – Participants; Published protocol (doi.org/10.3310/nihropenres.13512.2)
Variables	7	Clearly define all outcomes, exposures, predictors, potential confounders, and effect modifiers. Give diagnostic criteria, if applicable	Methods – Procedures; Published protocol (doi.org/10.3310/nihropenres.13512.2)
Data sources/ measurement	8	For each variable of interest, give sources of data and details of methods of assessment (measurement). Describe comparability of assessment methods if there is more than one group	Methods – Procedures; Published protocol (doi.org/10.3310/nihropenres.13512.2)
Bias	9	Describe any efforts to address potential sources of bias	Published protocol (doi.org/10.3310/nihropenres.13512.2)
Study size	10	Explain how the study size was arrived at	Methods – Statistical analysis
Quantitative variables	11	Explain how quantitative variables were handled in the analyses. If applicable, describe which groupings were chosen and why	Methods – Outcomes; Methods – Statistical analysis
Statistical methods	12	(*a*) Describe all statistical methods, including those used to control for confounding	Methods – Statistical analysis
		(*b*) Describe any methods used to examine subgroups and interactions	Methods – Statistical analysis
		(*c*) Explain how missing data were addressed	Methods – Statistical analysis
		(*d*) If applicable, explain how loss to follow-up was addressed	Methods – Statistical analysis
		(*e*) Describe any sensitivity analyses	Methods – Outcomes; Methods – Statistical analysis
**Results**			
Participants	13*	(a) Report numbers of individuals at each stage of study—eg numbers potentially eligible, examined for eligibility, confirmed eligible, included in the study, completing follow-up, and analysed	Figure 1; Figure S14
		(b) Give reasons for non-participation at each stage	Figure 1; Figure S14
		(c) Consider use of a flow diagram	Figure 1; Figure S14
Descriptive data	14*	(a) Give characteristics of study participants (eg demographic, clinical, social) and information on exposures and potential confounders	Table 1.
		(b) Indicate number of participants with missing data for each variable of interest	Table 1; Tables S5-S9
		(c) Summarise follow-up time (eg, average and total amount)	Results
Outcome data	15*	Report numbers of outcome events or summary measures over time	Results
Main results	16	(*a*) Give unadjusted estimates and, if applicable, confounder-adjusted estimates and their precision (eg, 95% confidence interval). Make clear which confounders were adjusted for and why they were included	Results; Figures 2-3; Tables 2-3
		(*b*) Report category boundaries when continuous variables were categorized	Results; Figures 2-3; Tables 2-3
		(*c*) If relevant, consider translating estimates of relative risk into absolute risk for a meaningful time period	Results
Other analyses	17	Report other analyses done—eg analyses of subgroups and interactions, and sensitivity analyses	Tables S1-14; Figures S1-S5
**Discussion**			
Key results	18	Summarise key results with reference to study objectives	Discussion
Limitations	19	Discuss limitations of the study, taking into account sources of potential bias or imprecision. Discuss both direction and magnitude of any potential bias	Discussion
Interpretation	20	Give a cautious overall interpretation of results considering objectives, limitations, multiplicity of analyses, results from similar studies, and other relevant evidence	Discussion
Generalisability	21	Discuss the generalisability (external validity) of the study results	Discussion
**Other information**			
Funding	22	Give the source of funding and the role of the funders for the present study and, if applicable, for the original study on which the present article is based	Grant information

Site characteristics

Recruitment site	Characteristics
Chiradzulu District Hospital (CDH), Chiradzulu, Malawi	Government District Hospital<br><br>Southern Malawi Region<br><br>Secondary care hospital. Receives direct admissions from community and referrals from primary health centres.<br><br>300 bed capacity. No intensive care unit<br><br>Catchment area: 389,928 population
Queen Elizabeth Central Hospital, Blantyre, Malawi	Government (public) Central Hospital.<br><br>Southern Malawi Region<br><br>Tertiary referral hospital. Also acts as secondary level service due to absence of other secondary health centres in Blantyre. The hospital provides medical care for patients presenting directly from the community, those referred from local primary health centres or from secondary health centres in the Southern Malawi region<br><br>1350 bed capacity. 10 bed intensive care unit<br><br>Catchment area:<br>Blantyre city: 871,776 Southern region population: 7,912,347
Hai District Hospital (HDH), Hai, Tanzania	Government District Hospital<br>Kilimanjaro Region in the Northern Zone of Tanzania<br><br>Secondary care hospital. Receives direct admissions from community and referrals from primary health centres in the district.<br><br>140 bed capacity. No intensive care unit.<br><br>Catchment area population: 240,999 (Hai District population, Tanzania Census, 2022)
Muhimbili National Referral Hospital (MNH), Dar es Salaam, Tanzania	Government National Referral hospital.<br><br>Dar es Salaam City, main commercial hub of Tanzania.<br><br>National Referral Hospital largest public hospital in Tanzania, serving as the top referral centre for all hospitals and the majority of patients are referred from hospitals across the country. A minority of cases will be severe acute illness in community patients needing immediate emergency care. The hospital does not accept referrals from primary health centres, unless there are special emergency circumstances for primary health centres close to MNH.<br><br>1500 bed capacity. 73 beds intensive care unit<br><br>Catchment area: National Hospital thus serves a population of 62 million.

Case definitions

Hypertension definition

- A diagnosis of hypertension was confirmed if ⩾2 sequential blood pressure measurements are recorded as SBP ⩾140mmHg and/or DBP ⩾90mmHg on two separate days, among patients who do not have diabetes, chronic kidney disease or who are at high risk of cardiovascular disease (CVD).
- The WHO package of essential noncommunicable (PEN) and hypertension treatment guidelines report additional targets of systolic blood pressure (SBP) <130 mmHg and diastolic blood pressure (DBP) <80 mmHg as recommended for patients with diabetes, chronic kidney disease (CKD) or at high risk of CVD. HIV is increasingly recognised as an independent risk factor for CVD.[1] As such, a diagnosis of hypertension was also be confirmed if ⩾2 blood pressure measurements are recorded as SBP ⩾130mmHg and/or DBP ⩾80mmHg on two separate days, among patients with diabetes, CKD, HIV, stroke or ischaemic heart disease.
- BP measurement followed WHO guidance.[2]
- In instances of a missing second BP measurement, we also reviewed clinical records, with consideration of treatment, patient history and clinically recorded diagnoses (see Tables S5-6 for details).

Table S 1 Hypertension control

Category	Good control	Grade 1	Grade 2	Hypertensive crisis
**Systolic BP (mmHg)**	<140<br><130 (in presence of CVD risk factors)	140-159	≥160	≥180
**Diastolic BP (mmHg)**	<90<br><80 (in presence of CVD risk factors)	90-99	≥100	≥120

Diabetes mellitus definition

Diabetes was defined in line with WHO PENS guidance.[3] For operationalisation the following approaches will be used:

- All participants were tested for HBA1c irrespective of diagnostic status.
- Participants without a prior diagnosis were considered diabetic if HBA1c is ≥6.5% (or ≥48mmol/L depending on locally preferred units). HBA1c was used rather than fasting blood glucose (FBG) as FBG can be difficult to interpret in hospitalized patients with acute illness.
- In instances of missing HBA1c results, we also reviewed clinical records, with consideration of treatment, patient history and clinically recorded diagnoses (see Table S7 for details)

Table S 2 Diabetes control

Control	Very good control	Good control	Poor control	Very poor control
***HbA1c level, %**	<6.5%	6.5-6.9%	≥7-7.9 %	≥8%

*HbA1c used to ascertain disease control among patients with diabetes mellitus [4-6].

HIV definition

HIV was diagnosed in line with the WHO Consolidated Guidelines on HIV Testing Services.[7]

- HIV point-of-care rapid antibody blood testing will be required for patients without a pre-existing HIV diagnosis.
- HIV rapid antibody testing is widely available and is routinely conducted within study hospitals.
- HIV viral load testing was conducted only in participants who have consented for inclusion and who are positive for HIV-infection.
- We also reviewed clinical records, with consideration of treatment, patient history and clinically recorded diagnoses (see Table S8 for details).

Table S 3 HIV control

Viral load	Undetectable	Controlled	Uncontrolled	Very poor control	High infectivity
Copies/mL	<50	≥50-199	≥200-999	≥1000-9999	≥10,000

HIV viral load used as the biomarker for disease control.[8-11]

Chronic kidney disease (CKD) definition

We used the 2021 CKD-EPI equation [12] to estimate glomerular filtration rate (eGFR) in mL/min/1.73 m^2 which requires sex, age and creatinine values for the calculation. Creatinine will be measured using a point of care test. CKD will be defined in line with KDIGO[13,14] guidance:

- Persistently reduced eGFR (< 60ml/min/1.73m^2) at both day 0 and day 90.
- Unavailable modalities of the KDIGO [15] guidelines will be excluded. For example, albumin : creatinine ratio estimation is not available. We will not use urinalysis (to assess proteinuria) on the CKD classification as this is the least preferred option for assessing kidney disease[13]; due to poor sensitivity and high false-discovery rate,[16] which are further attenuated in acute illness.[15]
- In instances of missing creatinine levels at baseline or day 90, we also reviewed clinical records, with consideration of patient history and clinically recorded diagnoses (in line with KDIGO guidelines[13,14]; see Table S9 for details)

Table S 4 Chronic kidney disease (CKD) stage

Stage	eGFR
**3a**	45-59
**3b**	30-44
**4**	15-29
**5**	<15

CKD using the eGFR, calculated using the CKD-EPI 2021 formula [17].

Depression

All participants answered questions on PHQ2. If participants had a score of ≥3 on the PHQ2, they proceeded to answer questions from the full PHQ9.[18]

PHQ9:
0 "No / minimal depression"
5 "Mild depression"
10 "Moderate depression"
15 "Moderately severe depression"
20 "Severe depression"

*PHQ9 score ≥10 was classified as "depression" [18] as the cut off for treatment

Multilink cohort study: algorithms for coding clinical diagnoses.

Table S 5 Hypertension. Hypertension as single disease

	**Diagnostic step**	**n, positive**	**n, negative**
**1. BP proven Hypertension (gold-standard diagnosis)**	1. Hypertension in absence of cardiovascular risk factors: two sequential readings to support BP. (Can use D90 if only one reading as an in-patient).<br>(SBP ≥ 140 / DBP ≥ 90)	274	0
	2. Single BP reading: SBP ≥160 / DBP ≥ 100	122	0
	3. Negative (in absence of CV risk factors: first two sequential readings from the dataset below threshold levels,<br>AND not prescribed antihypertensive medications<br>AND patient states they do not have hypertension	0	618
**2. BP measurement x 1 AND Clinical information x1**	4. Measured BP on D0 supports diagnosis (SBP ≥ 140 / DBP ≥ 90) and either:<br>- participant states they have HTN<br>- prescribed antihypertensives	126	0
**3. Clinical information x2**	5. Any two of:<br>- clinical diagnosis;<br>- patient states they have hypertension;<br>- prescribed antihypertensive.	204	0
**4. Clinical information x1**	6. Any one of:<br>- Clinical diagnosis<br>- patient prescribed meds*	11	0
	7. Patient states they have or do not have hypertension	44	8
	**Total, n (%)**	**781 (55.5%**	**626 (44.5%)**

*Patients prescribed ACE-inhibitors or angiotensin receptor blockers in cases of heart failure have not been classified as hypertensive at step 6.

Table S 6 Hypertension. Hypertension diagnosis in presence of cardiovascular disease.

Cardiovascular Risk factors = diabetes mellitus, chronic kidney infection, infection with HIV, stroke, ischaemic heart disease

	**Diagnostic step**	**n, positive**	**n, negative**
**1. BP proven Hypertension (gold-standard diagnosis)**	1. Hypertension in presence of cardiovascular risk factors: two sequential readings to support BP. (Can use D90 if only one reading as an in-patient).<br>(SBP ≥ 130 / DBP ≥ 80)	442	0
	2. Single BP reading: SBP ≥160 / DBP ≥ 100	82	0
	3. Negative (in absence of CV risk factors: first two sequential readings from the dataset below threshold levels,<br>AND not prescribed antihypertensive medications<br>AND patient states they do not have hypertension.	0	573
**2. BP measurement x 1 AND Clinical information x1**	4. Measured BP on D0 supports diagnosis (SBP ≥ 130 / DBP ≥ 80) and either:<br>- participant states they have HTN<br>- prescribed antihypertensives	127	0
**3. Clinical information x2**	5. Any two of:<br>- clinical diagnosis;<br>- patient states they have hypertension;<br>- prescribed antihypertensive.	138	0
**4. Clinical information x1**	6. Any one of:<br>- Clinical diagnosis<br>- patient prescribed meds*	5	0
	7. Patient states they have or do not have hypertension	33	7
	**Total, n (%)**	**827 (58.8%)**	**580 (41.2%)**

*Patients prescribed ACE-inhibitors or angiotensin receptor blockers in cases of heart failure have not been classified as hypertensive at step 6.

Table S 7 Diabetes

	Diagnostic step	n, positive	n, negative
**1. Measured HbA1c proven diabetes mellitus (Gold-standard diagnosis)**	1. Positive if HbA1c ≥48mmol/l (≥6.5%)	444	0
	2. Negative if HbA1c < 42mmol/L and not taking diabetic medications	0	655
	3. Diabetes: Pre-diabetes threshold level (HbA1c 42-47mmol/L) & on diabetic medications (prescribed on any follow up)	32	0
**2. Clinical information x2**	4. Positive if patient states they are diabetic and on diabetic medications	48	0
	5. Positive if patient states they are diabetic and random blood sugar high (≥ 11.1)	6	0
	6. Positive if there is a recorded clinical diagnosis of diabetes and on diabetic meds (± patients says they are not diabetic)	9	0
	7. Negative if patients says they are not diabetic and random blood sugar <11.1	0	192
**3. Clinical information x1**	8. Patient prescribed diabetic medications	0	0
	9. Clinical diagnosis	6	0
	10. Patient states they have or do not have diabetes	2	13
	**Total, n (%)**	**547 (38.9%)**	**860 (61.1%)**

Table S 8 HIV

	Diagnostic step	n, positive	n, negative
**1. POC or laboratory proven HIV**	1. HIV status confirmed by study tests (RDT): positive and negative	10	331
	2. HIV positive if viral load taken (and > than undetectable) (baseline or d90)	161	0
	3. HIV positive if viral load taken (and undetectable at baseline or d90) and on ARV (ARV on any follow up)	142	0
**2. Clinical information x2**	4. HIV positive if patient states positive & patient is on ARVs.	50	0
	5. HIV positive if patient is prescribed ARVs: prescribed ever & clinical diagnosis of HIV (on any follow-up)	1	0
**3. Clinical information x1**	6. HIV positive if prescribed ARVs	0	0
	7. HIV positive if clinical diagnosis of HIV (at any stage)	1	0
	8. HIV positive if patient states HIV positive (baseline, discharge, d90)	3	0
	9. If empty cell, HIV negative if patient states negative (baseline, discharge, d90). Test result < 12 months	0	531
	10. If empty cell, HIV negative if patient states negative (test date ≥12 months or unknown)	0	168
	**Total, n(%)**	**368 (26.2%)**	**1030** (73.2%)

*9 (0.6%) unclassified

Table S 9 CKD

	Diagnostic step	n, positive	n, negative
**1. Gold-standard diagnosis**	1. Positive if 2 x eGFR < 60 at D0 & D90	74	0
	2. Negative if either D0 or d90 eGFR ≥60	0	883
**2. Laboratory info x1 + Clinical information x1**	3. Positive if patient states they have CKD, corroborated by eGFR < 60 on admission.	186	0
	4. Positive if clinical diagnosis of CKD, corroborated by eGFR < 60 on admission	69	0
**3. Clinical information x2**	5. Positive if clinical diagnosis of CKD & patient states they have CKD	7	0
**4. Clinical information x1**	6. Positive if clinical diagnosis of CKD	2	0
	7. Patient states they have or do not have CKD.	2	184
	**Total, n (%)**	**340 (24.2%)**	**1067 (75.8%)**

Heart failure, stroke, chronic liver disease, ischaemic heart disease, chronic obstructive pulmonary disease

Step 1

Diagnosis positive if clinical diagnosis on discharge (in-patient outcome CRF)
Diagnosis negative if no record of diagnosis made on discharge (in-patient outcome CRF)

Steps 2-4

If no discharge form, clinical diagnosis present on D7 (step 2), D5 (step 3), D2 (step 4).

Step 5

Patient reported existing diagnosis

Frailty

We assessed frailty using the clinical frailty scale (CFS); defined as CFS ≥5.[19]

Disability

Disability was determined according to the Washington Group-Short Set (WG-SS) on Functioning,[20] as anyone having at least 'a lot of difficulty' on at least one of the six questions (on vision, hearing, mobility, self-care, cognition and communication). For the Cognition section of the WG-SS, to avoid over-duplication of questions, we used responses from the PHQ-9 question on cognition: "Do you have trouble concentrating on things, such as reading the newspaper or watching television?" A response of not at all in the PHQ-9 was coded as "no difficulty" in the WG-SS; "several days" to "some symptoms"; "more than half the days" to "a lot of difficulty"; "nearly every day" to "cannot do at all".

Table S 10 Existing evidence to underpin selection of functional tools used within the cohort study.

Tool	Validation
PHQ9	doi.org/10.1186/s12888-019-2062-2 (Malawi)<br>doi: 10.1016/j.npbr.2018.11.002 (Tanzania)<br>doi: https://doi.org/10.1136/bmj.n2183 (In-patient settings)
Clinical frailty scale (CFS)	doi: 10.1093/qjmed/hcv066 (in-patient settings, not in Africa)
Washington Group-Short Set (WG-SS) on Functioning	https://www.washingtongroup-disability.com/ (International use)<br>doi.org/10.12688/wellcomeopenres.15196.5 (Malawi)
EQ5D-5L	doi: 10.4314/mmj.v29i2.2 (Malawi)<br>euroqol.org/

Figure S 1 Prevalence and characteristics of multimorbidity among people admitted to hospital in Queen Elizabeth Central Hospital, Malawi

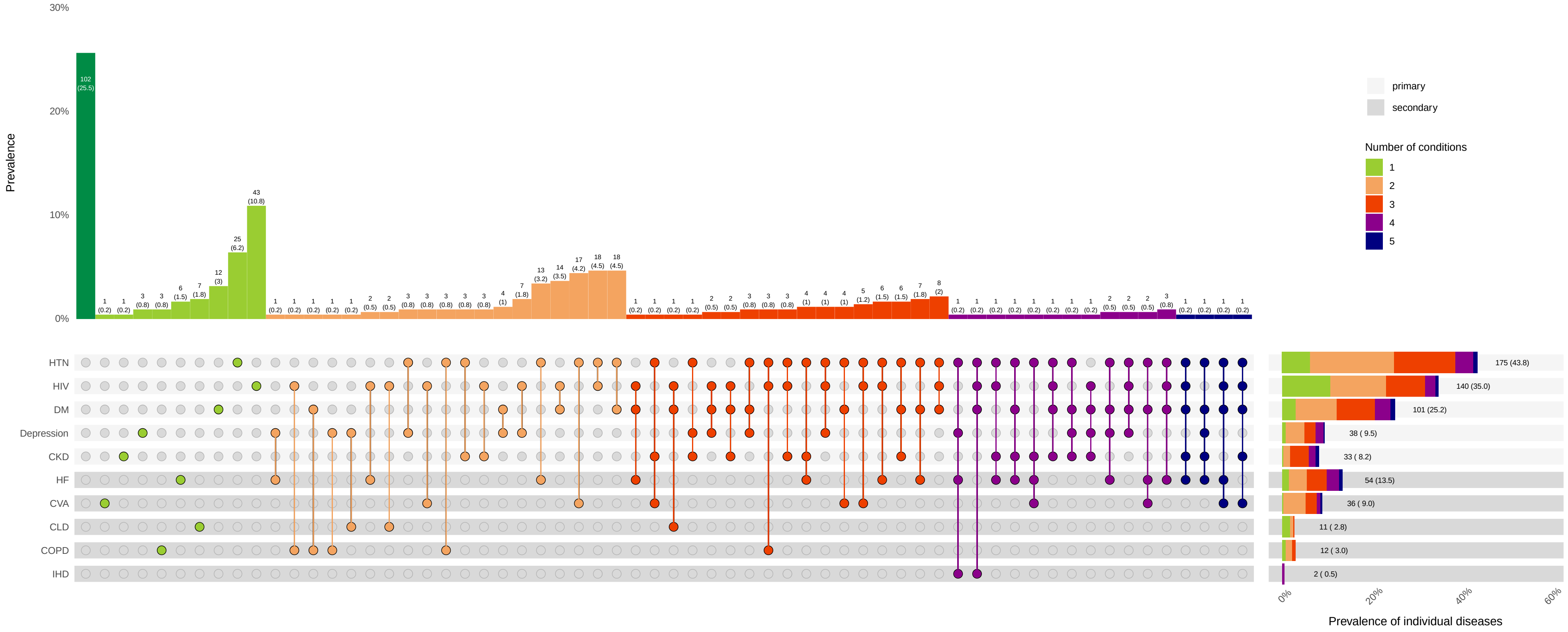

Figure S 2 Prevalence and characteristics of multimorbidity among people admitted to hospital in Chiradzulu District Hospital**, Malawi**

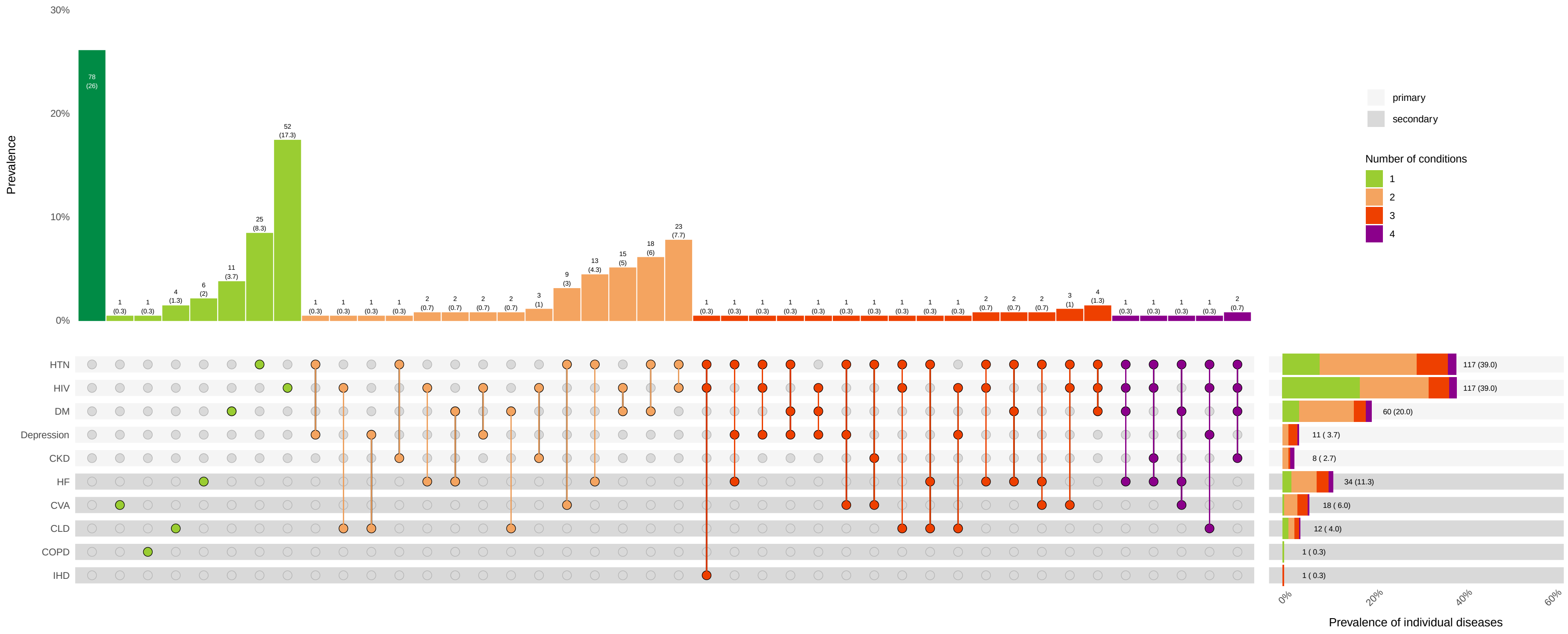

Figure S 3 Prevalence and characteristics of multimorbidity among people admitted to hospital in Hai District Hospital, Tanzania

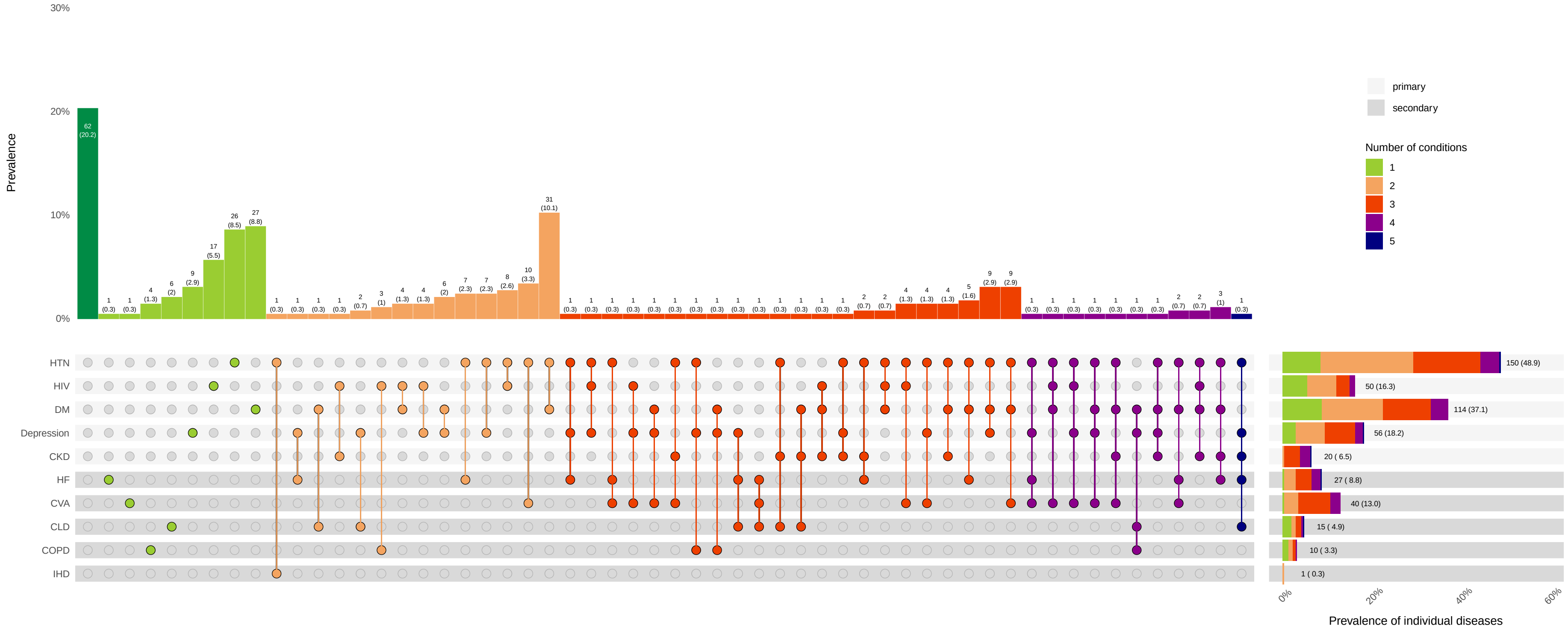

Figure S 4 Prevalence and characteristics of multimorbidity among people admitted to hospital in Muhimbili National Hospital, Tanzania

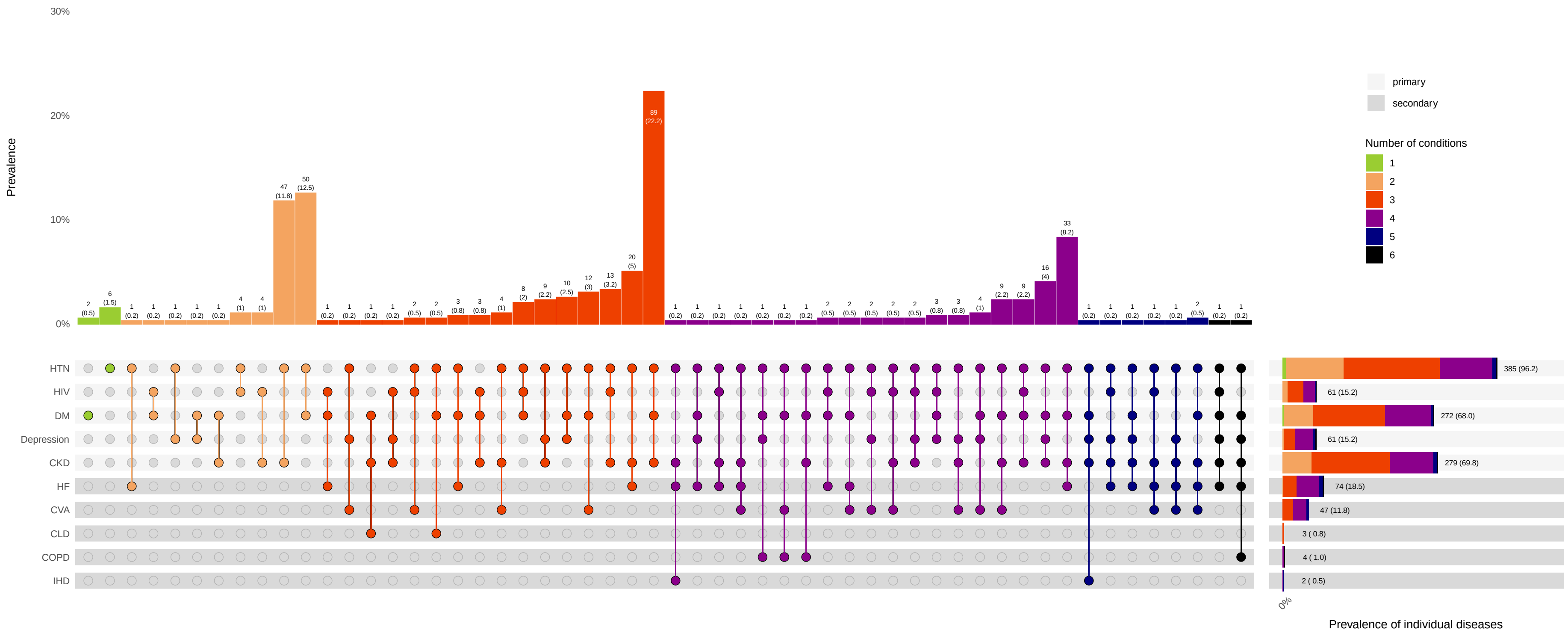

Figure S 5 Prevalence of Multimorbidity by site.

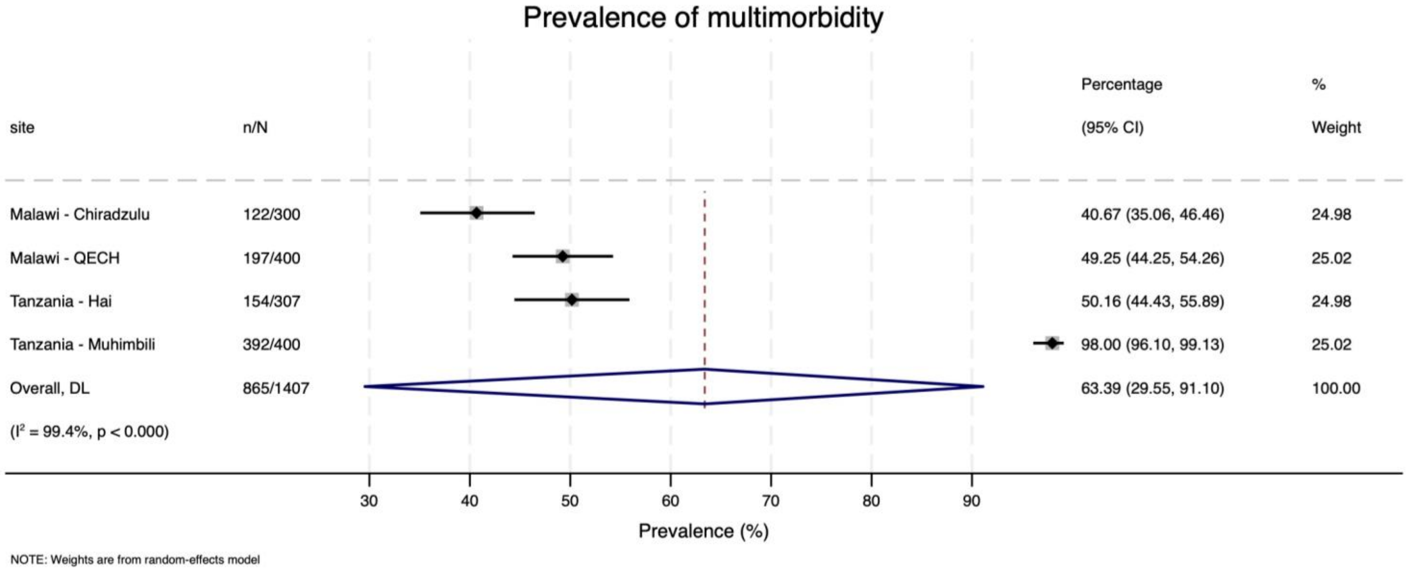

Figure S 6 Prevalence of Multimorbidity across three sites.

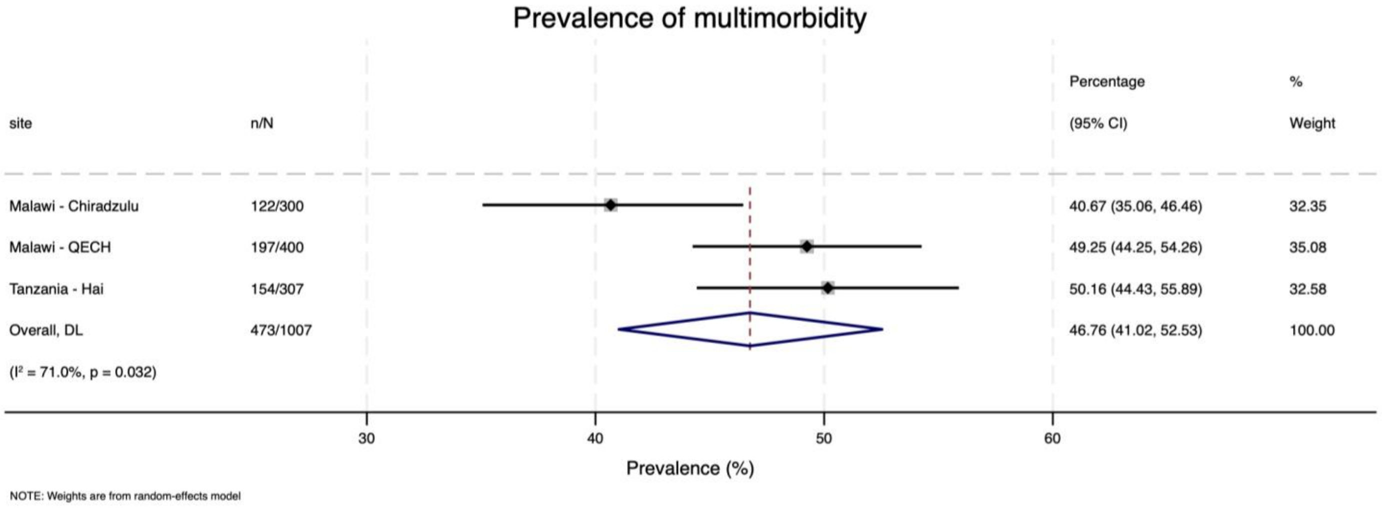

Figure S 7 Prevalence of conditions diagnosed through study procedures: all sites

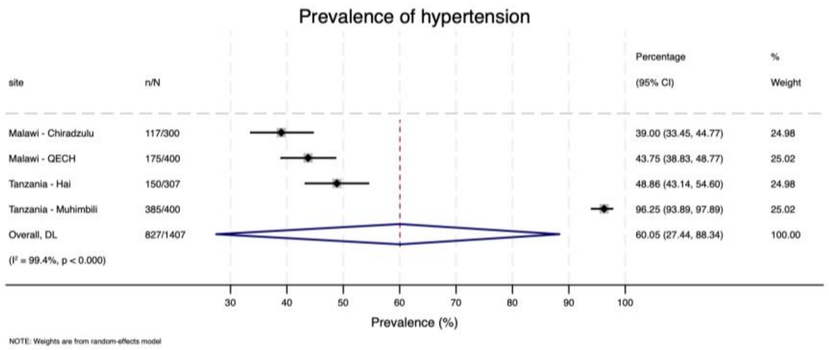

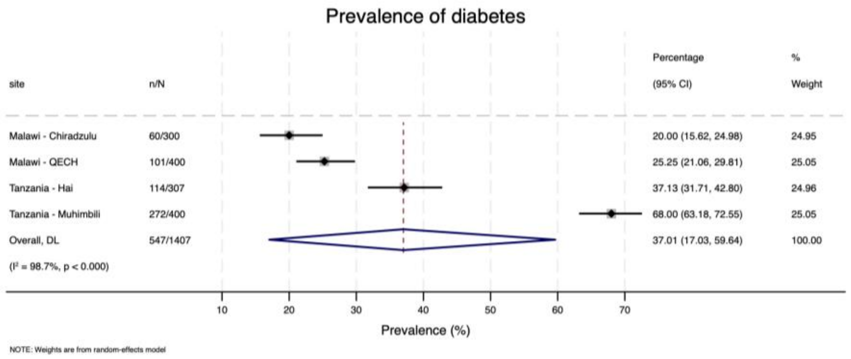

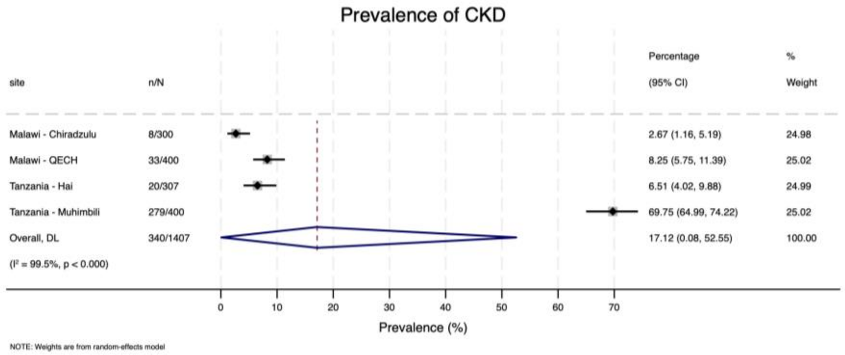

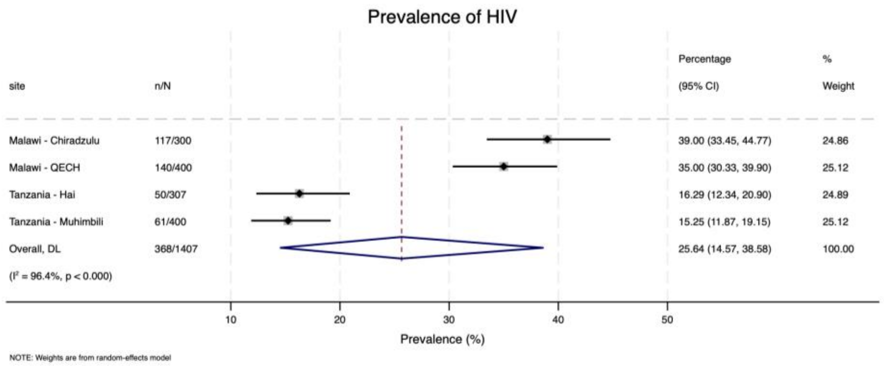

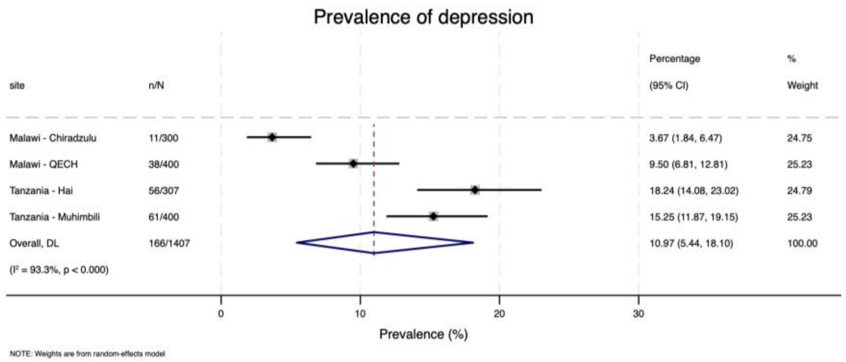

Figure S 8 Prevalence of conditions diagnosed through study procedures: three sites

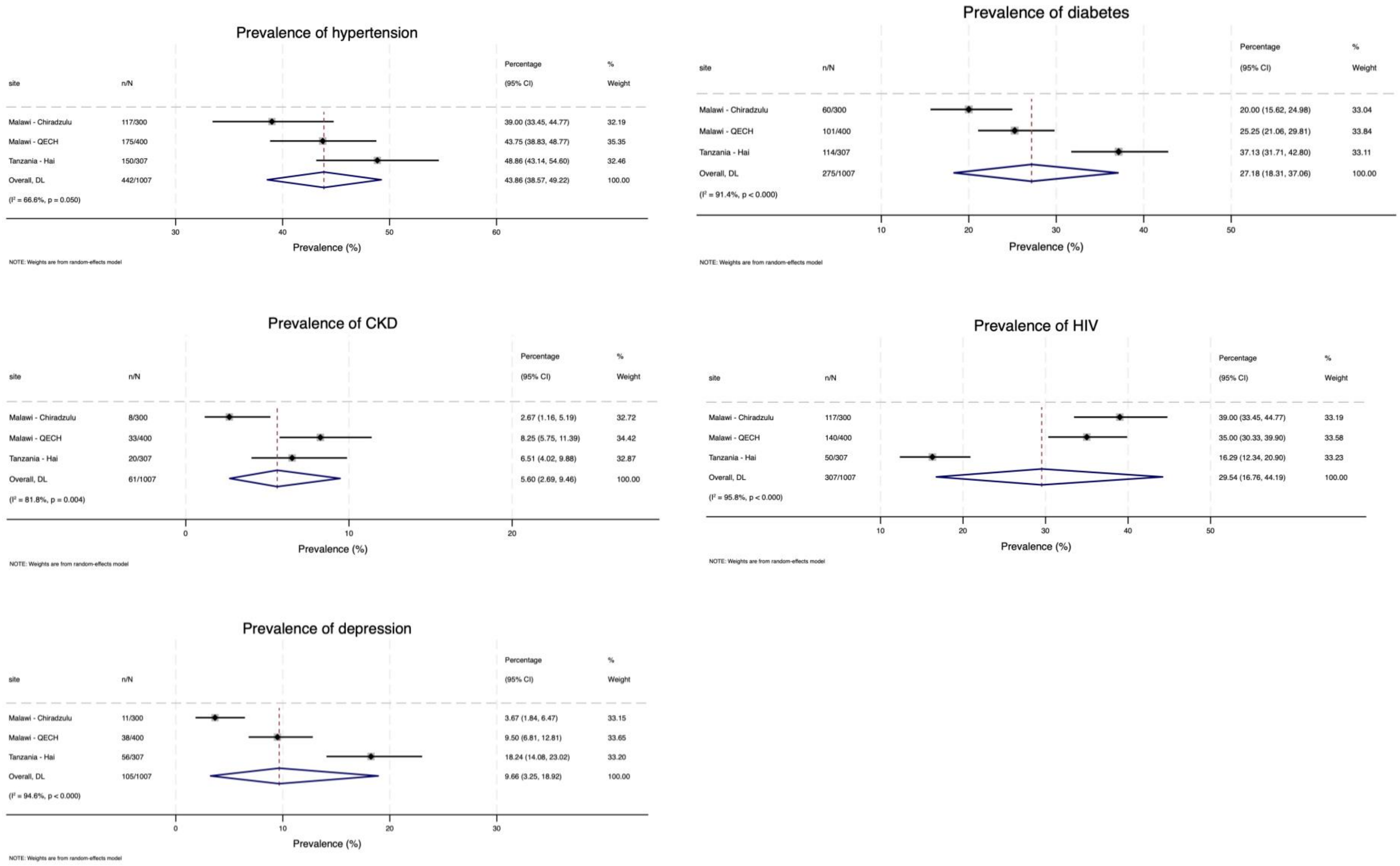

Figure S 9 Prevalence of clinically diagnosed conditions: all sites

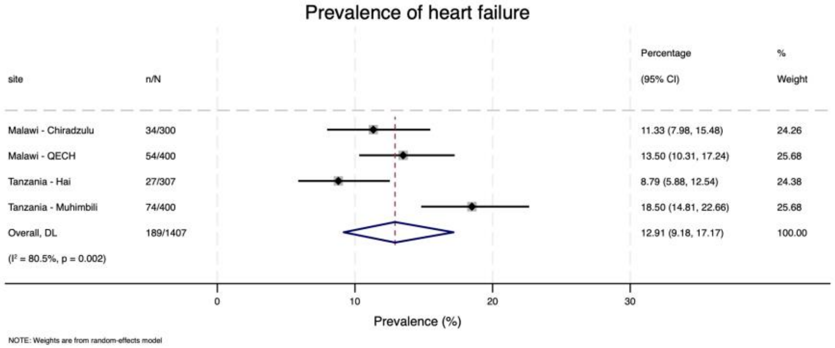

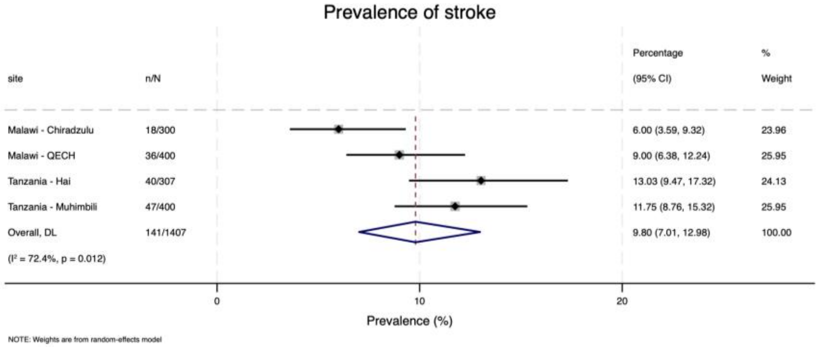

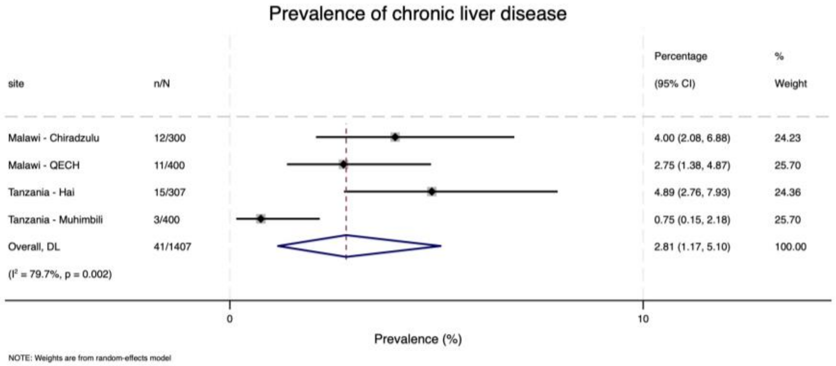

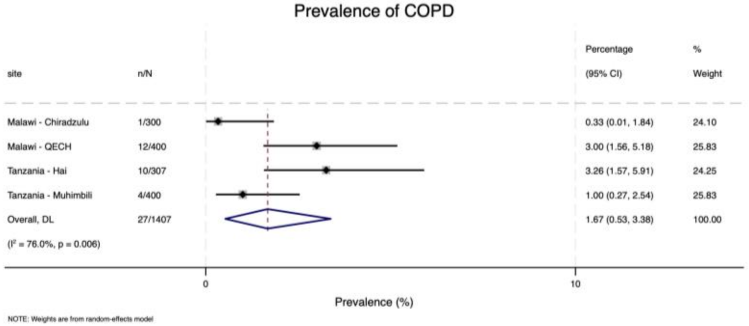

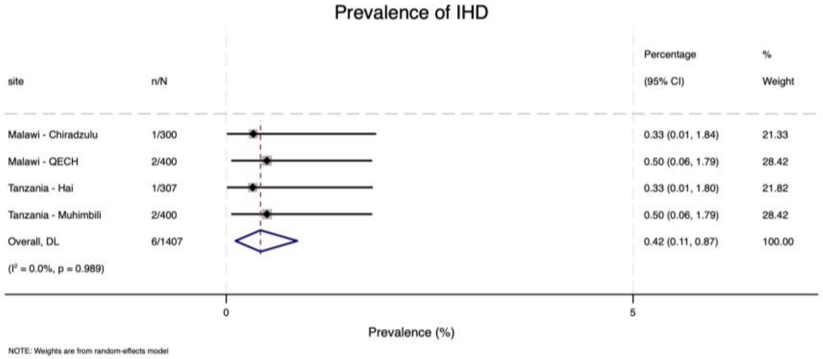

Figure S 10 Prevalence of clinically diagnosed conditions: three sites

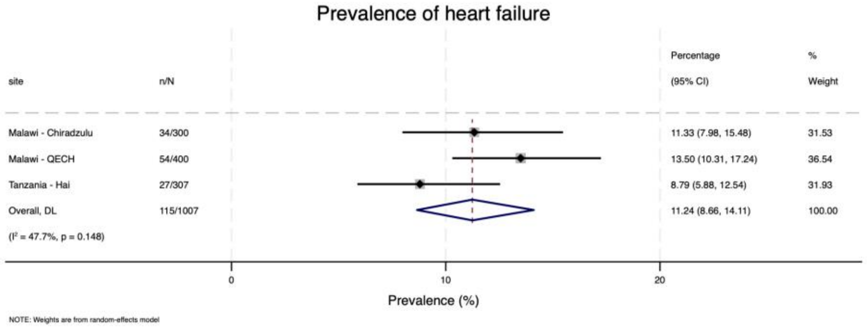

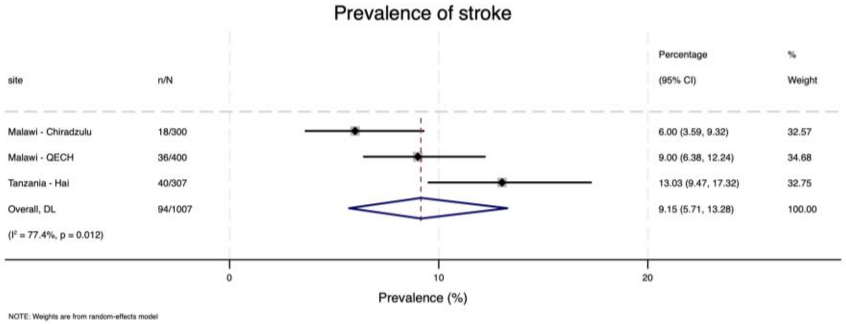

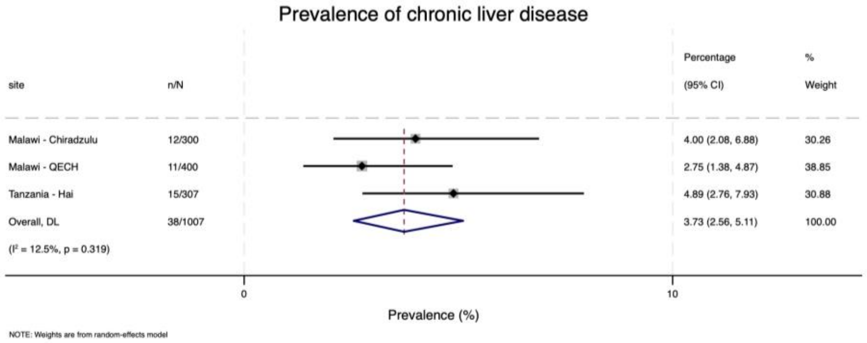

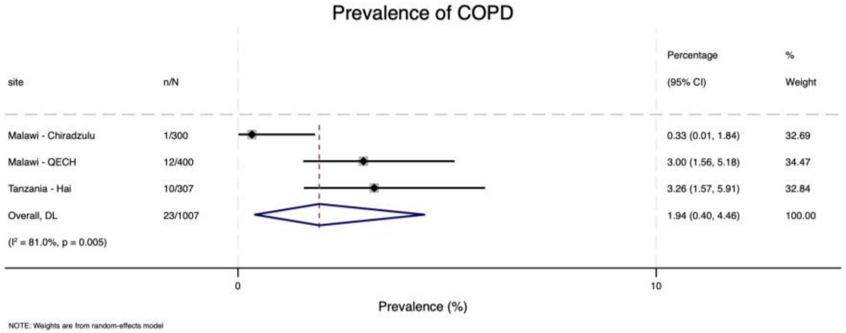

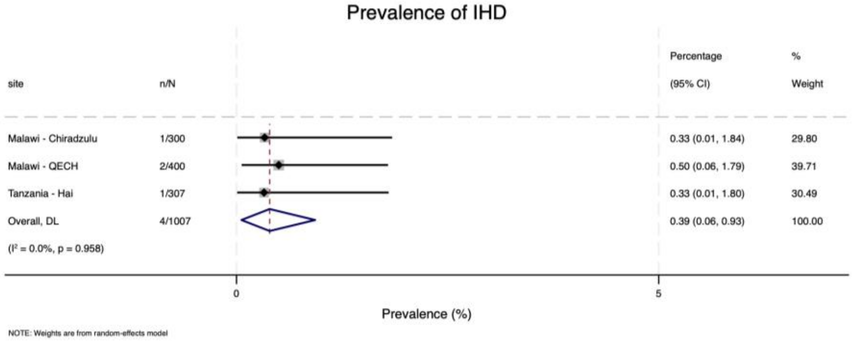

Table S 11 Functional and mental health measures

	QECH	Chiradzulu	Hai	Muhimbili
N	400	300	307	400
**Washington Group – Short Set on Functioning**				
*Disability**				
With disability	184 (46.0%)	93 (31.0%)	137 (44.6%)	87 (21.8%)
Without disability	216 (54.0%)	207 (69.0%)	170 (55.4%)	313 (78.2%)
Vision				
No difficulty	337 (84.2%)	284 (94.7%)	220 (71.7%)	368 (92.0%)
Some difficulty	58 (14.5%)	12 (4.0%)	71 (23.1%)	28 (7.0%)
A lot of difficulty	3 (0.8%)	2 (0.7%)	9 (2.9%)	2 (0.5%)
Cannot do at all	2 (0.5%)	2 (0.7%)	7 (2.3%)	2 (0.5%)
Hearing				
No difficulty	387 (96.8%)	286 (95.3%)	271 (88.3%)	382 (95.5%)
Some difficulty	11 (2.8%)	14 (4.7%)	27 (8.8%)	16 (4.0%)
A lot of difficulty	0 (0.0%)	0 (0.0%)	2 (0.7%)	1 (0.2%)
Cannot do at all	2 (0.5%)	0 (0.0%)	7 (2.3%)	1 (0.2%)
Mobility				
No difficulty	95 (23.8%)	89 (29.7%)	155 (50.5%)	124 (31.0%)
Some difficulty	168 (42.0%)	130 (43.3%)	58 (18.9%)	228 (57.0%)
A lot of difficulty	83 (20.8%)	59 (19.7%)	34 (11.1%)	22 (5.5%)
Cannot do at all	54 (13.5%)	22 (7.3%)	60 (19.5%)	26 (6.5%)
Cognition#				
No difficulty	10 (2.5%)	3 (1.0%)	29 (9.4%)	13 (3.2%)
Some difficulty	32 (8.0%)	1 (0.3%)	7 (2.3%)	14 (3.5%)
A lot of difficulty	23 (5.8%)	1 (0.3%)	11 (3.6%)	11 (2.8%)
Cannot do at all	11 (2.8%)	6 (2.0%)	17 (5.5%)	28 (7.0%)
Missing	324 (81.0%)	289 (96.3%)	243 (79.2%)	334 (83.5%)
Self care				
No difficulty	164 (41.0%)	208 (69.3%)	166 (54.1%)	268 (67.0%)
Some difficulty	149 (37.2%)	49 (16.3%)	55 (17.9%)	99 (24.8%)
A lot of difficulty	45 (11.2%)	35 (11.7%)	15 (4.9%)	19 (4.8%)
Cannot do at all	42 (10.5%)	8 (2.7%)	71 (23.1%)	14 (3.5%)
Communication				
No difficulty	333 (83.2%)	257 (85.7%)	231 (75.2%)	319 (79.8%)
Some difficulty	35 (8.8%)	36 (12.0%)	35 (11.4%)	60 (15.0%)
A lot of difficulty	12 (3.0%)	5 (1.7%)	16 (5.2%)	8 (2.0%)
Cannot do at all	20 (5.0%)	2 (0.7%)	25 (8.1%)	13 (3.2%)
**Clinical frailty scale**				
Clinical Frailty				
Not frail (CFS<5)	193 (48.2%)	190 (63.3%)	222 (72.3%)	56 (14.0%)
Frail (CFS 5-6)	161 (40.2%)	59 (19.7%)	43 (14.0%)	105 (26.2%)
Severely frail (CFS ≥7)	46 (11.5%)	51 (17.0%)	42 (13.7%)	239 (59.8%)
**EQ5D-5L**				
Mobility				
No problems	69 (17.2%)	51 (17.0%)	100 (32.6%)	31 (7.8%)
Slight problems	124 (31.0%)	124 (41.3%)	39 (12.7%)	130 (32.5%)
Moderate problems	113 (28.2%)	45 (15.0%)	50 (16.3%)	120 (30.0%)
Severe problems	28 (7.0%)	61 (20.3%)	18 (5.9%)	84 (21.0%)
Unable to do	66 (16.5%)	19 (6.3%)	100 (32.6%)	35 (8.8%)
Self care				
No problems	95 (23.8%)	72 (24.0%)	118 (38.4%)	37 (9.2%)
Slight problems	140 (35.0%)	125 (41.7%)	32 (10.4%)	131 (32.8%)
Moderate problems	87 (21.8%)	48 (16.0%)	40 (13.0%)	124 (31.0%)
Severe problems	25 (6.2%)	44 (14.7%)	13 (4.2%)	87 (21.8%)
Unable to do	53 (13.2%)	11 (3.7%)	104 (33.9%)	21 (5.2%)
Usual activities				
No problems	34 (8.5%)	51 (17.0%)	56 (18.2%)	25 (6.2%)
Slight problems	112 (28.0%)	132 (44.0%)	23 (7.5%)	148 (37.0%)
Moderate problems	144 (36.0%)	61 (20.3%)	41 (13.4%)	102 (25.5%)
Severe problems	47 (11.8%)	41 (13.7%)	10 (3.3%)	90 (22.5%)
Unable to do	63 (15.8%)	15 (5.0%)	177 (57.7%)	35 (8.8%)
Pain/Discomfort				
No symptoms	101 (25.2%)	35 (11.7%)	82 (26.7%)	72 (18.0%)
Slight symptoms	123 (30.8%)	83 (27.7%)	65 (21.2%)	105 (26.2%)

Moderate symptoms	141 (35.2%)	113 (37.7%)	110 (35.8%)	140 (35.0%)
Severe symptoms	26 (6.5%)	64 (21.3%)	41 (13.4%)	75 (18.8%)
Extreme symptoms	9 (2.2%)	5 (1.7%)	9 (2.9%)	8 (2.0%)
Anxiety/Depression				
No symptoms	170 (42.5%)	94 (31.3%)	173 (56.4%)	116 (29.0%)
Slight symptoms	157 (39.2%)	103 (34.3%)	41 (13.4%)	116 (29.0%)
Moderate symptoms	58 (14.5%)	73 (24.3%)	64 (20.8%)	76 (19.0%)
Severe symptoms	14 (3.5%)	28 (9.3%)	20 (6.5%)	69 (17.2%)
Extreme symptoms	1 (0.2%)	2 (0.7%)	9 (2.9%)	23 (5.8%)
Mean EQ5D VAS [SD]	55.2 [17.7]	62.9 [13.6]	57.7 [17.5]	61.4 [16.4]
**PHQ-9**				
Depression severity				
No / minimal depression	326 (81.5%)	289 (96.3%)	243 (79.2%)	335 (83.8%)
Moderate depression	29 (7.2%)	4 (1.3%)	24 (7.8%)	25 (6.2%)
Moderately severe depression	5 (1.2%)	3 (1.0%)	23 (7.5%)	12 (3.0%)
Severe depression	4 (1.0%)	4 (1.3%)	9 (2.9%)	24 (6.0%)
Mild depression	36 (9.0%)	0 (0.0%)	8 (2.6%)	4 (1.0%)

* Disability is determined, according to the Washington Group-Short Set (WG-SS) on Functioning, as anyone having at least ‘a lot of difficulty’ on at least one of the six questions (on vision, hearing, mobility, self-care, cognition and communication)

For the Cognition section of the WG-SS, to avoid over-duplication of questions, we used responses from the PHQ-9 question on cognition: “Do you have trouble concentrating on things, such as reading the newspaper or watching television?” A response of not at all in the PHQ-9 was coded as “no difficulty” in the WG-SS; “several days” to “some symptoms”; “more than half the days” to “a lot of difficulty”; “nearly every day” to “cannot do at all”

Table S 12 Association between number of long-term conditions, and: age; sex; and severity of illness (universal vital assessment).

	Total, N=1407	No long-term conditions, N = 242	Single long-term condition, N = 300	≥2 long-term conditions, N = 865	p-value
Age group					
18-29	190 (13.5%)	86 (35.5%)	59 (19.7%)	45 (5.2%)	
30-39	191 (13.6%)	57 (23.6%)	51 (17.0%)	83 (9.6%)	
40-49	274 (19.5%)	43 (17.8%)	65 (21.7%)	166 (19.2%)	
50-59	239 (17.0%)	20 (8.3%)	39 (13.0%)	180 (20.8%)	
60-69	244 (17.3%)	14 (5.8%)	38 (12.7%)	192 (22.2%)	
70-79	170 (12.1%)	11 (4.5%)	28 (9.3%)	131 (15.1%)	
80-89	63 (4.5%)	6 (2.5%)	11 (3.7%)	46 (5.3%)	
≥90	36 (2.6%)	5 (2.1%)	9 (3.0%)	22 (2.5%)	
Mean age [SD]	52.3 [18.4]	39.8 [18.1]	48.4 [19.3]	57.1 [16.1]	<0.0001§
Sex					
Female	657 (46.7%)	91 (37.6%)	147 (49.0%)	419 (48.4%)	
Male	750 (53.3%)	151 (62.4%)	153 (51.0%)	446 (51.6%)	0.01*
Universal vital assessment					
Low risk (UVA 0–1)	661 (47.0%)	186 (76.9%)	132 (44.0%)	343 (39.7%)	
Medium risk (UVA 2–4)	580 (41.2%)	50 (20.7%)	135 (45.0%)	395 (45.7%)	
High risk (UVA >4)	166 (11.8%)	6 (2.5%)	33 (11.0%)	127 (14.7%)	<0.0001§

§ Kruskal-Wallis test using continuous variable for age and UVA
*Chi-squared test.

Table S 13 Association between number of long-term conditions and: frailty, disability.

	Total, N=1407	No long-term conditions, N = 242	Single long-term condition, N = 300	≥2 long-term conditions, N = 865	p-value
Clinical Frailty Scale (CFS)					
Not frail (CFS ≤4)	661 (47.0%)	177 (73.1%)	201 (67.0%)	283 (32.7%)	
Frail (CFS 5-6)	368 (26.2%)	52 (21.5%)	60 (20.0%)	256 (29.6%)	
Severely frail (CFS ≥ 7)	378 (26.9%)	13 (5.4%)	39 (13.0%)	326 (37.7%)	<0.0001§
Disability					
Without disability	906 (64.4%)	186 (76.9%)	203 (67.7%)	517 (59.8%)	
With disability	501 (35.6%)	56 (21.1%)	97 (32.3%)	348 (40.2%)	<0.0001*

§ Kruskal-Wallis test using original clinical frailty scale
*Chi-squared test.

Figure S 11 Prevalence of multimorbidity, by age, sex, universal vital assessment (UVA) and clinical frailty scale (CFS).

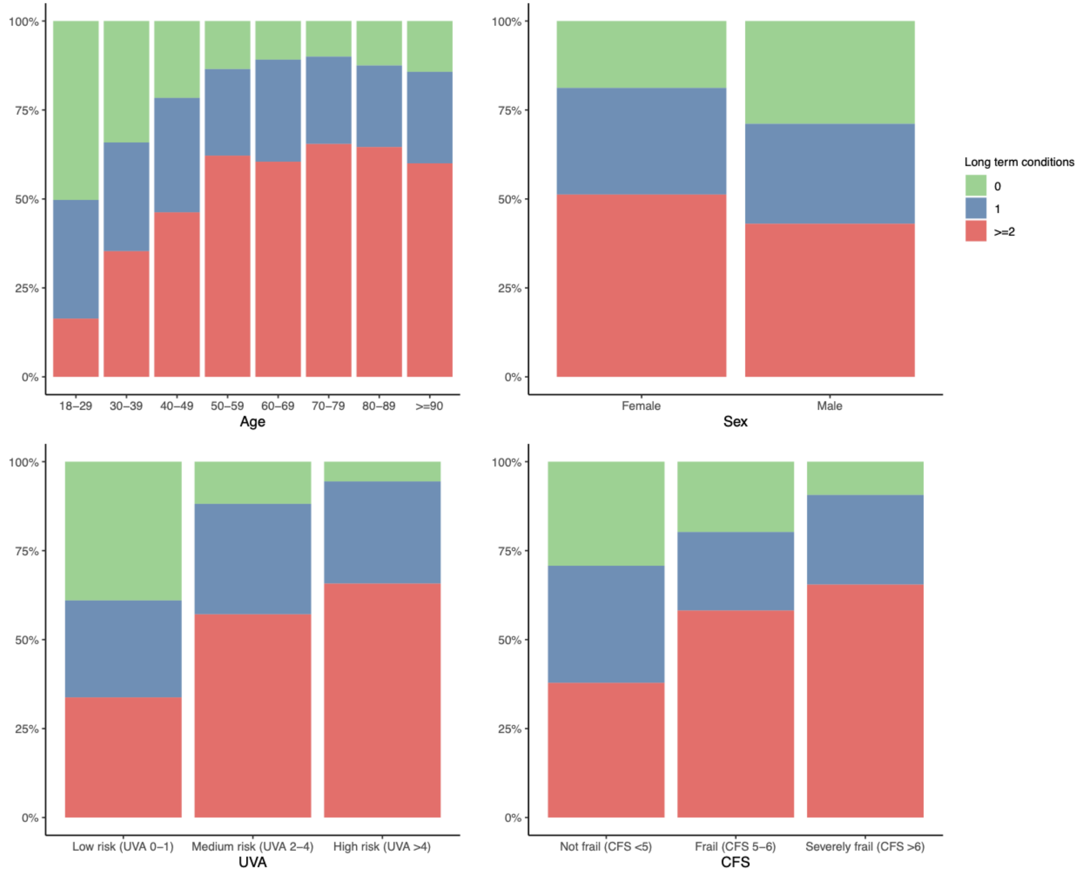

Table S 14 Association between number of long-term conditions and HRQoL health utility at baseline

	Total, N=1407	No long-term conditions, N = 242	1 long-term condition, N = 300	≥2 long-term conditions, N = 865	Single vs no long-term conditions: p-value		≥2 vs no long-term conditions: p-value		≥2 vs single long-term conditions: p-value	
					Coefficient (95% CI)	p-value	Coefficient (95% CI)	p-value	Coefficient (95% CI)	p-value
Baseline median HRQoL health utility (IQR)	0.492 (0.05, 0.667)	0.633 (0.310, 0.799)	0.557 (0.140, 0.730)	0.402 (-0.037, 0.644)	NA	0.005§	NA	<0.0001§	NA	<0.0001§
					0.01 (-0.02, 0.04)	0.36*	-0.02 (-0.05, 0.01)	0.13*	-0.04 (-0.06, -0.01)	0.005*
Sensitivity analysis: depression excluded from multimorbidity disease count										
	N=1407	*N = 254*	*N=331*	*N = 822*						
Baseline median HRQoL health utility (IQR)	0.492 (0.05, 0.667)	*0.623 (0.288, 0.796)*	*0.534 (0.050, 0.717)*	*0.419 (-0.036, 0.655)*	*NA*	*0.0005§*	*NA*	*<0.0001§*	*NA*	*0.0008§*
					0.01 (-0.02, 0.04)	*0.61**	*0.00 (-0.03, 0.03)*	*0.93**	*-0.01 (-0.03, 0.02)*	*0.62**

§ Mann-Whitney U test
*GLM (gamma distribution) multivariable model with adjustments for age, sex, universal vital assessment, and site.
NA: not applicable

Table S 15 Association between number of long-term conditions and HRQoL health utility at the final observation

	Total, N=649	No long-term conditions, N = 169	1 long-term condition, N = 163	≥2 long-term conditions, N = 317	1 vs no long-term conditions: p-value		≥2 vs no long-term conditions: p-value		≥2 vs 1 long-term conditions: p-value	
					Coefficient (95% CI)	p-value	Coefficient (95% CI)	p-value	Coefficient (95% CI)	p-value
Final observation median HRQoL health utility (IQR)	0.940 (0.749, 1.00)	1.00 (0.873, 1.00)	1.00 (0859, 1.00)	0.858 (0.667, 1.00)	NA	0.04§	NA	<0.0001§	NA	<0.0001§
					-0.01 (-0.03, 0.02)	0.70*	-0.03 (-0.05, -0.01)	0.006*	-0.03 (-0.03, -0.01)	0.01*
					-0.01 (-0.03, 0.02)	*0.71‡*	*-0.03 (-0.06, -0.01)*	*0.005‡*	*-0.03 (-0.05, -0.01)*	*0.01‡*
Sensitivity analysis: depression excluded from multimorbidity disease count										
	N = 649	*N = 174*	*N = 173*	*N=302*						
Final observation median HRQoL health utility (IQR)	*0.940 (0.749, 1.00)*	*1.00 (0.873, 1.00)*	*1.00 (0.799, 1.00)*	*0.860 (0.667, 1.00)*	*NA*	*0.006§*	*NA*	*<0.0001§*	*NA*	*<0.0001§*
					-0.01 (-0.03, 0.01)	*0.440**	*-0.03 (-0.05, -0.01)*	*0.012**	*-0.02 (-0.04, -0.00)*	*0.068**

§Mann-Whitney U test
*GLM (Gamma distribution) multivariable model with adjustments for age, sex, number of days between admission, and site
Italic font reflect sensitivity analyses
‡ Multiple imputation estimates based on GLM (Gamma distribution) multivariable model with adjustments for age, sex, site and number of days between admission and final observation. Multiple imputation with chained equations (MICE; *m* = 10 imputed datasets). Number of observations: n=837. The MI analyses were conducted using all the variables in the analysis model (HRQoL utility at day 90, number of long-term conditions, age, sex, site, number of days between admission and final observation), as well as the auxiliary variables (HRQoL utility at baseline, disability at baseline, clinical frailty, mid-upper arm circumference, and individual diseases: hypertension, diabetes, CKD, depression, HIV, heart failure, stroke, chronic liver disease, ischaemic heart disease, COPD). The variables with imputed data included: HRQoL utility at the final observation, mid-upper arm circumference. The imputation was conducted using STATA 18 (*mi impute chained* command); 10 datasets were imputed and with a burn-in of 10 iterations.
NA: not applicable

Table S 16 Association between 90-day mortality and: frailty, disability.

	Survived	Died	Odds ratio (95% CI)	p-value*
Clinical Frailty Scale (CFS)				
Not frail (CFS ≤4)	512/625 (81.9%)	113/625 (18.1%)	Ref	Ref
Frail (CFS 5-6)	216/345 (62.6%)	129/345 (37.4%)	2.7 (2.0-3.6)	<0.0001
Severely frail (CFS ≥ 7)	143/347 (41.2%)	204/347 (58.8%)	6.4 (4.8-8.7)	<0.0001
Disability				
Without disability	614/834 (73.6%)	220/834 (26.4%)	Ref	Ref
With disability	258/483 (53.2%)	226/483 (46.8.3%)	2.5 (1.93-3.10)	<0.0001

*Univariable logistic regression.

Table S 17 Univariable and multivariable Cox regression analyses

	Total	No long-term conditions	Single long-term condition	≥2 long-term conditions	Univariable analyses		Multivariable analyses	
					Single vs no long-term conditions: HR (95%CI) §	≥2 vs no long-term conditions: HR (95%CI) §	Single vs no long-term conditions: HR (95%CI) *	≥2 vs no long-term conditions: OR (95%CI) *
Survival throughout follow up								
Censored (LTFU)	90/1407 (6.4%)							
Survived to day 90	871/1317 (66.1%)	199/230 (86.5%)	203/283 (71.7%)	469/804 (58.3%)	Ref	Ref	Ref	Ref
Died by day 90	446/1317 (33.9%)	31/230 (13.5%)	80/283 (28.3%)	335/804 (41.7%)	2.0 (1.36-2.79)	3.1 (2.23-4.19)	1.5 (1.01-2.11)	1.5 (1.05-2.11)

§ Univariable Cox regression

*Multivariable Cox regression with adjustments for age, sex, universal vital assessment, and site

Table S 18 In-patient, D30 and D90 outcomes of patients with multimorbidity, vs single long term and no long-term conditions

	Total	No long-term conditions	Single long-term condition	≥2 long-term conditions	Univariable analyses		Multivariable analyses	
					Single vs no long-term conditions: OR (95%CI) §	≥2 vs no long-term conditions: OR (95%CI) §	Single vs no long-term conditions: OR (95%CI) *	≥2 vs no long-term conditions: OR (95%CI) *
In-patient outcome								
Survived	1125/1312 (85.7%)	221/235 (94.0%)	259/290 (89.3%)	645/787 (82.0%)				
Died	187/1312 (14.3%)	14/235 (6.0%)	31/290 (10.7%)	142/787 (18.0%)	1.9 (0.98-3.64)	3.5 (1.97-6.14)	1.2 (0.62-2.49)	1.41 (0.74-2.70)
Day 30 outcome								
Survived	1044/1366 (76.4%)	213/234 (91.0%)	238/290 (82.1%)	593/842 (70.4%)				
Died	322/1366 (23.6%)	21/234 (9.0%)	52/290 (17.9%)	249/842 (29.6%)	2.2 (1.19-3.80)	4.3 (2.65-6.82)	1.5 (0.85-2.64)	1.60 (0.94-2.73)
Day 90 outcome								
Survived	871/1317 (66.1%)	199/230 (86.5%)	203/283 (71.7%)	469/804 (58.3%)				
Died	446/1317 (33.9%)	31/230 (13.5%)	80/283 (28.3%)	335/804 (41.7%)	2.5 (1.60-4.00)	4.6 (3.06-6.87)	1.7 (1.08-2.82)	2.0 (1.24-3.08)

§ Univariable logistic regression
*Multivariable logistic regression with adjustments for age, sex, universal vital assessment, and site

Table S 19 Sensitivity analyses showing best- and worst-case survival outcomes at D90 comparing patients with multimorbidity, vs single long-term and no long-term conditions

	Total	No long-term conditions	Single long-term condition	≥2 long-term conditions	Single vs no long-term conditions: OR (95%CI) §	≥2 vs no long-term conditions: OR (95%CI) §	Single vs no long-term conditions: OR (95%CI) *	≥2 vs no long-term conditions: OR (95%CI) *
BEST CASE								
Day 90 outcome								
Survived	961/1407 (68.3%)	211/242 (87.2%)	220/300 (73.3%)	530/865 (61.3%)				
Died	446/1407 (31.7%)	31/242 (12.8%)	80/300 (26.7%)	335/865 (38.7%)	2.5 (1.60-3.90)	4.3 (1.57-3.90)	1.8 (1.11-2.89)	2.1 (1.33-3.27)
WORST CASE								
Day 90 outcome								
Survived	871/1407 (61.9%)	199/242 (82.2%)	203/300 (67.7%)	469/865 (54.2%)				
Died	536/1407 (38.1%)	43/242 (17.8%)	97/300 (32.3%)	396/865 (45.8%)	2.2 (1.47-3.33)	3.9 (2.74-5.58)	1.6 (1.07-2.51)	1.7 (1.10-2.50)

§ Univariable logistic regression
*Multivariable logistic regression with adjustments for age, sex, universal vital assessment, and site

Table S 20 HbA1c at baseline and at day 90.

	Baseline HbA1c (n=1304)	Baseline HBA1C in Day 90 survivors (n=785)	Day 90 HbA1c (n=618)	p-value*
Median HbA1c [IQR]	40 [34-54]	39 [33-50]	36 [30-45]	<0.0001

*Wilcoxon signed-rank test: paired test between participants with both baseline and day 90 HbA1c measurement.

Figure S 12 Kaplan Meier survival plots for participants with 0, 1, 2, 3 and ≥4 long-term conditions

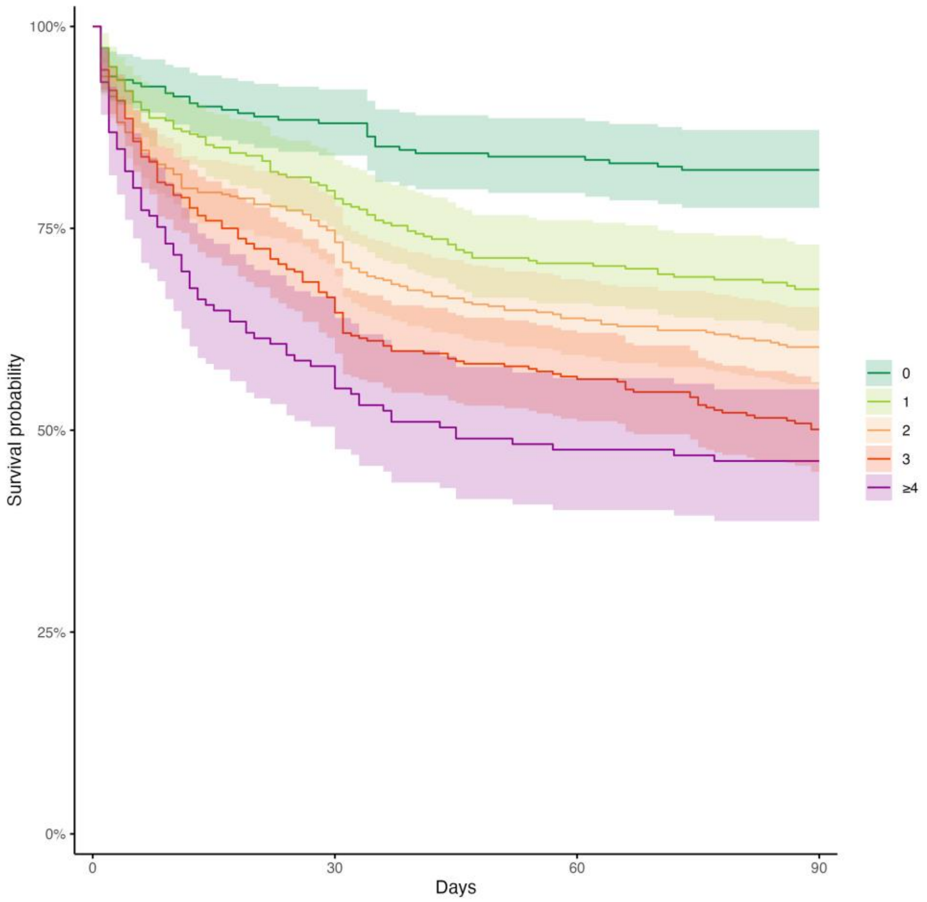

Table S 21 Table comparing age between consented participants and participants who declined consent.

	Consented (n = 1437)§	Declined consent (n = 102)	p-value*
Mean age [SD]	52.0 [18.4]	52.5 [18.3]	0.82

*ANOVA test
§Note while 1437 consented, a baseline assessment was not collected on 30 participants and subsequent analyses were therefore not possible for these participants (see Figure 1)

Table S 22 Table comparing demographics between patients followed up at day 90 and those lost to follow-up.

	Followed-up (n = 1317)	Lost to follow-up (n = 90)
Mean age [SD]	52.0 [18.4]	56.9 [17.8]
Female	609 (46.2%)	48 (53.3%)
Male	708 (53.8%)	42 (46.7%)
Site		
Chiradzulu District Hospital, Malawi	300 (22.8%)	0 (0%)
Queen Elizabeth Central Hospital, Malawi	398 (30.2%)	2 (2.2%)
Hai District Hospital, Tanzania	269 (20.4%)	38 (42.2%)
Muhimbili National Hospital, Tanzania	350 (26.6%)	50 (55.6%)
HIV	361/1309 (27.6%)	7/89 (7.9%)
Hypertension	763 (57.9%)	64 (71.1%)
Diabetes	500 (38.0%)	47 (52.2%)
Chronic kidney disease	309 (23.5%)	31 (34.4%)
Depression	154 (11.7%)	12 (13.3%)
Heart failure	183/1300 (14.1%)	6/82 (7.3%)
Stroke	132/1300 (10.2%)	9/83 (10.8%)
Chronic obstructive pulmonary disease	26/1301 (2.0%)	1/82 (1.2%)
Ischaemic heart disease	6/1300 (0.5%)	0/82 (0.0%)
Chronic liver disease	37/1300 (2.8%)	4/82 (4.9%)
Clinical Frailty		
Not frail (CFS<5)	625 (47.5%)	36 (40.0%)
Frail (CFS 5-6)	345 (26.2%)	23 (25.6%)
Severely frail (CFS ≥7)	347 (26.3%)	31 (34.4%)
Disability	483 (36.7%)	18 (20.0%)
Universal vital assessment (UVA) score		
Low risk (UVA 0–1)	601 (45.6%)	60 (66.7%)
Medium risk (UVA 2–4)	554 (42.1%)	26 (28.9%)
High risk (UVA >4)	162 (12.3%)	4 (4.4%)
Current tobacco smoker	88 (6.7%)	2 (2.2%)
Current alcohol use	226/1310 (17.3%)	14 (15.6%)
Number of long-term conditions		
0	230 (17.5%)	12 (13.3%)
1	283 (21.5%)	17 (18.9%)
2+	804 (61.0%)	61 (67.8%)

Table S 23 Table comparing demographics between participants randomised into cost/income sub-cohort and those not randomised.

	Cost/income sub-cohort	
	**No (n=944)**	**Yes (n=463)**
Mean age [SD]	52.4 [18.4]	52.0 [18.4]
Female	438/944 (46.4%)	219/463 (47.3%)
Male	506/944 (53.6%)	244/463 (52.7%)
Site		
Chiradzulu District Hospital, Malawi	208/944 (22.0%)	92/463 (19.9%)
Queen Elizabeth Central Hospital, Malawi	259/944 (27.4%)	141/463 (30.5%)
Hai District Hospital, Tanzania	204/944 (21.6%)	103/463 (22.3%
Muhimbili National Hospital, Tanzania	273/944 (28.9%)	127/463 (27.4%)
HIV	234/938 (24.9%)	134/460 (29.1%)
Hypertension	566/944 (60.0%)	261/463 (56.4%)
Diabetes	378/944 (40.0%)	169/463 (36.5%)
Chronic kidney disease	231/944 (24.5%)	109/463 (23.5%)
Depression	113/944 (12.0%)	53/463 (11.4%)
Heart failure	125/931 (13.4%)	64/451 (14.2%)
Stroke	88/932 (9.4%)	53/451 (11.8%)
COPD	22/932 (2.4%)	5/451 (1.1%)
Ischaemic heart disease	4/931 (0.4%)	2/451 (0.4%)
Chronic liver disease	24/931 (2.6%)	17/451 (3.8%)
Clinical Frailty		
Not frail (CFS<5)	435/944 (46.1%)	226/463 (48.8%)
Frail (CFS 5-6)	255/944 (27.0%)	113/463 (24.4%)
Severely frail (CFS ≥7)	254/944 (26.9%)	124/463 (26.8%)
Disability	332/944 (35.2%)	169/463 (36.5%)
Universal vital assessment score		
Low risk (UVA 0–1)	448/944 (47.5%)	213/463 (46.0%)
Medium risk (UVA 2–4)	388/944 (41.1%)	192/463 (41.5%)
High risk (UVA >4)	108/944 (11.4%)	58/463 (12.5%)
Current tobacco smoker	55/944 (5.8%)	35/463 (7.6%)
Current alcohol use	164/938 (17.5%)	76/462 (16.5%)
Long term conditions		
0	172/944 (18.2%)	70/463 (15.1%)
1	188/944 (19.9%)	112/463 (24.2%)
2+	584/944 (61.9%)	281/463 (60.7%)
In-patient mortality	124/879 (14.1%)	63/433 (14.5%)
Day 30 mortality	217/912 (23.8%)	105/454 (23.1%)
Day 90 mortality	293/879 (33.3%)	153/438 (34.9%)

Table S 24 Variable missingness in multivariable analyses: survival analyses, HRQoL, cost.

Covariate	Number (%) missing
**Survival analyses:**	
number LTFU (censored)	90/1407 (6.4%)
**HRQoL analyses**	
Baseline HRQoL	0/1407 (0%)
Final observation	188/837 (22.5%)
**Cost analyses**	0/463 (0%)
**Co-variates common to all models:**	
Number of long-term conditions	0 (0%)
Age	0 (%)
Sex	0 (0%)
UVA	0 (0%)

Table S 25 Participant cost and income effects (mean), Malawi and Tanzania (USD$)

	Number of long-term conditions					
	0		1		≥2	
	Mean (95% CI)	%	Mean (95% CI)	%	Mean (95% CI)	%
MALAWI	N=52		N=79		N=102	
**Participant costs**						
Medical costs[a]	3.78 (-1.16 - 8.73)	8.6	3.36 (0.65 - 6.06)	7.0	11.73 (-1.46 - 24.93)	8.2
Non-Medical costs[b]	18.64 (10.21 - 27.07)	77.0	12.67 (10.29 - 15.04)	80.8	22.54 (16.16 - 28.92)	79.8
Indirect costs[c]	3.81 (1.21 - 6.42)	14.5	17.83 (1.30 - 34.35)	12.2	22.52 (2.28 - 42.75)	12.0
Total costs	26.24 (15.92- 36.55)	100	33.85 (16.45 - 51.25)	100	56.79 (27.44 - 86.13)	100
**Personal monthly income**						
Pre-hospitalisation	49.37 (29.20 - 69.55)		48.85 (34.64 - 63.06)		73.30 (48.08 - 98.51)	
Post-hospitalisation	27.89 (16.88 - 38.91)		33.67 (20.81 - 46.54)		43.65 (27.56 - 59.75)	
Individual income loss*	26.73 (7.95 - 45.51)		17.51 (8.03 - 26.00)		37.78 (14.60 - 60.95)	
TANZANIA	N=18		N=33		N=179	
**Participant costs**						
Medical costs[a]	54.69 (26.55- 82.84)	63.4	65.59 (35.08- 96.10)	62.1	205.56 (152.52- 258.59)	72.0
Non-Medical costs[b]	13.16 (6.54- 19.78)	25.1	12.87 (9.34- 16.41)	25.3	9.19 (7.27 - 11.11)	8.0
Indirect costs[c]	13.81 (-0.44- 28.07)	11.5	146.15 (-133.39 - 425.68)	12.6	92.0 (55.5 - 128.5)	19.1
Total cost	81.67(47.56 - 115.78)	100	224.61 (-60.47 - 509.69)	100	306.8 (242.13- 371.42)	100
**Personal monthly income**						
Pre-hospitalisation	33.78 (16.43 - 51.13)		145.76 (-72.93 - 364.45)		115.77 (91.47 - 140.06)	
Post-hospitalisation	26.34 (10.07 - 42.61)		24.96 (10.49 - 39.44)		100.84 (77.74 - 123.95)	
Individual income loss[§]	11.70 (0 .77 - 22.62)		24.96 (10.49 - 39.44)		20.29 (9.28 - 31.31)	

[a] Medical costs comprise medications, tests and medical supplies.

[b] Non-medical costs comprise transport and food for participants and their guardians.

[c] Indirect costs are estimated for each patient based on the reported number of workdays lost multiplied by the reported mean monthly pre-illness income, divided by 22 days.

Table S 26 Difference in costs between participants with 0, 1 and ≥2 long-term conditions. Results from multivariable GLM model

	1 vs 0 long-term conditions		≥2 vs 0 long-term conditions		≥2 vs 1 long-term conditions	
	p-value	Relative effect (95% CI)	p-value	Relative effect (95% CI)	p-value	Relative effect (95% CI)
MALAWI						
**Participant costs (USD$)**						
Medical costs [a]	0.831	0.83 (0.15 - 4.48)	0.205	3.04 (0.54 - 16.96)	0.150	1.20 (0.22 - 6.47)
Non-medical costs [b]	0.655	0.90 (0.55 - 1.45)	0.151	1.43 (0.88 - 2.35)	0.023	1.60 (1.07 - 2.40)
Indirect costs [c]	0.03	10.40 (1.26 - 85.75)	0.038	15.98 (1.17 - 218.29)	0.500	1.54 (0.29 - 8.19)
**Personal monthly income**						
Pre-hospitalisation	0.393	1.28 (0.73 - 2.25)	0.059	1.72 (0.98 - 3.02)	0.221	1.35 (0.84 - 2.16)
Post-hospitalisation	0.058	1.96 (0.98 - 3.94)	0.010	2.61 (1.26 - 5.39)	0.320	1.33 (0.76 - 2.33)
Individual income loss	0.407	0.63 (0.22 - 1.86)	0.918	1.06 (0.36 - 3.11)	0.288	1.67 (0.65 - 4.29)
Household annual income	0.931	1.03 (0.54 - 1.95)	0.343	1.38 (0.71 - 2.69)	0.281	1.34 (0.79 - 2.29)
Catastrophic cost [d]	0.414	0.70 (0.30 - 1.65)	0.785	1.12 (0.49 - 2.57)	0.185	1.60 (0.80 - 3.23)
TANZANIA						
**Participant costs (USD$)**						
Medical costs [a]	0.379	1.40 (0.66 - 2.94)	<0.0001	3.93 (2.07 - 7.46)	<0.0001	2.81 (1.73 - 4.58)
Non-medical costs [b]	0.990	1.00 (0.46 - 2.14)	0.221	0.67 (0.35 - 1.27)	0.120	0.67 (0.40 - 1.11)
Indirect costs [c]	0.258	0.37 (0.07 - 2.07)	0.006	8.41 (1.82 - 38.91)	<0.0001	22.72 (7.04 - 73.28)
**Personal monthly income**						
Pre-hospitalisation	0.902	1.06 (0.39 - 2.87)	<0.0001	6.14 (2.54 - 14.84)	<0.0001	5.77 (2.99 - 11.15)
Post-hospitalisation	0.810	1.12 (0.43 - 2.91)	<0.0001	5.98 (2.48 - 14.44)	<0.0001	5.32 (2.77 - 10.22)
Individual income loss	0.836	0.66 (0.01 - 32.45)	0.578	2.38 (0.11 - 50.55)	0.314	3.60 (0.30 - 43.52)
Household annual income	0.395	1.49 (0.59 - 3.73)	<0.0001	7.15 (3.09 - 16.57)	<0.0001	4.80 (2.59 - 8.92)
Catastrophic cost [d]	0.324	1.87 (0.54 - 6.47)	0.199	0.50 (0.17 - 1.44)	0.003	0.27 (0.11 - 0.63)

Multivariable GLM model (Gamma distribution; link log function); adjusted by age, sex, and UVA. Relative effect reflects the adjusted relative difference in cost.

[a] Medical costs comprise medications, tests and medical supplies.

[b] Non-medical costs comprise transport and food for participants and their guardians.

[c] Indirect costs are estimated for each patient based on the reported number of workdays lost multiplied by the reported mean monthly pre-illness income, divided by 22 days. A high proportion of participants had no income, reflected within the reported values.

[d] Participants for whom total costs > 20% of annual reported household income (we collected monthly household income and multiplied by 12 months to estimate annual)

References

1. Feinstein MJ. HIV, Subclinical Cardiovascular Disease, and Clinical Progression: Insights From Immunologic Heterogeneity. *Jama* 2022; **328**(10): 931-2.
2. WHO. HEARTS Technical package for cardiovascular disease management in primary health care: evidence-based treatment protocols. Geneva, Switzerland: World Health Organization, 2018.
3. WHO. WHO package of essential noncommunicable (PEN) disease interventions for primary health care. 2020. https://www.who.int/publications/i/item/9789240009226 (accessed 12/06/2023.
4. Standards of medical care in diabetes--2013. *Diabetes Care* 2013; **36 Suppl 1**(Suppl 1): S11-66.
5. World Health Organization. Classification of Diabetes Mellitus. Geneva; 2019.
6. Federation ID. IDF Clinical Practice Recommendations for managing Type 2 Diabetes in Primary Care. Brussels; 2017.
7. WHO. WHO Consolidated Guidelines on HIV Testing Services 2019. https://www.who.int/teams/global-hiv-hepatitis-and-stis-programmes/hiv/testing-diagnostics/hiv-testing-services (accessed 12/06/2023.
8. World Health Organization. Consolidated Guidelines on HIV Prevention, Testing, Treatment, Service Delivery and Monitoring: Recommendations for a Public Health Approach. . Geneva; 2021.
9. Shoko C, Chikobvu D. A superiority of viral load over CD4 cell count when predicting mortality in HIV patients on therapy. *BMC Infectious Diseases* 2019; **19**(1): 169.
10. Kouanfack C, Mpoudi-Etame M, Omgba Bassega P, et al. Dolutegravir-Based or Low-Dose Efavirenz-Based Regimen for the Treatment of HIV-1. *N Engl J Med* 2019; **381**(9): 816-26.
11. Services UDoHH. Federally Approved Clinical Practice Guidelines for HIV/AIDS. 2023. https://clinicalinfo.hiv.gov/en/guidelines (accessed 24/11/2023.
12. Foundation NK. CKD-EPI Creatinine Equation (2021) 2021. https://www.kidney.org/professionals/kdoqi/gfr_calculator/formula (accessed 20/07/2023.
13. KDIGO Guidelines. 2023. https://kdigo.org/guidelines/ (accessed 20/07/2023.
14. Levin A, Ahmed SB, Carrero JJ, et al. Executive summary of the KDIGO 2024 Clinical Practice Guideline for the Evaluation and Management of Chronic Kidney Disease: known knowns and known unknowns. *Kidney Int* 2024; **105**(4): 684-701.
15. Group KDIGOC-MW. KDIGO clinical practice guideline for the diagnosis, evaluation, prevention, and treatment of Chronic Kidney Disease-Mineral and Bone Disorder (CKD-MBD). *Kidney international Supplement* 2009; (113): S1-S130.
16. Park JI, Baek H, Kim BR, Jung HH. Comparison of urine dipstick and albumin:creatinine ratio for chronic kidney disease screening: A population-based study. *PLoS One* 2017; **12**(2): e0171106.
17. Kidney Disease: Improving Global Outcomes Blood Pressure Work G. KDIGO 2021 Clinical Practice Guideline for the Management of Blood Pressure in Chronic Kidney Disease. *Kidney Int* 2021; **99**(3S): S1-S87.
18. Kroenke K, Spitzer RL, Williams JB. The PHQ-9: validity of a brief depression severity measure. *J Gen Intern Med* 2001; **16**(9): 606-13.
19. Wallis SJ, Wall J, Biram RW, Romero-Ortuno R. Association of the clinical frailty scale with hospital outcomes. *QJM* 2015; **108**(12): 943-9.
20. The Washington Group Short Set on Functioning (WG-SS). 2022. https://www.washingtongroup-disability.com (accessed 9 September 2024.
21. Yang F, Katumba KR, Roudijk B, et al. Developing the EQ-5D-5L Value Set for Uganda Using the 'Lite' Protocol. *Pharmacoeconomics* 2022; **40**(3): 309-21.
