# Supplementary material for: The burden of multimorbidity-associated acute hospital admissions in Malawi and Tanzania: a prospective multicentre cohort study
Source: Lancet Glob Health. 2025 Jun 25;13(7):e1279–90. doi: 10.1016/S2214-109X(25)00113-5 (PMC12208785; doi:10.1016/S2214-109X(25)00113-5)
Supplement: Equitable Partnership Declaration [file mmc4.pdf]

THE LANCET
Global Health

## Supplementary appendix 4

This Equitable Partnership Declaration (EPD) was submitted by the authors, and we reproduce it as supplied. It has not been peer reviewed. *The Lancet*'s editorial processes have not been applied to the EPD.

Supplement to: Spencer SA, Yongolo NM, Simiyu IG, et al. The burden of multimorbidity-associated acute hospital admissions in Malawi and Tanzania: a prospective multicentre cohort study. *Lancet Glob Health* 2025; **13:** e1279–90.

## Equitable Partnership Declaration

*Researcher considerations*

1. Please detail the involvement that researchers who are based in the region(s) of study had during a) study design; b) clinical study processes, such as processing blood samples, prescribing medication, or patient recruitment; c) data interpretation; and d) manuscript preparation, commenting on all aspects. If they were not involved in any of these aspects, please explain why.

   *This question is intended for international partnerships; if all your authors are based in the area of study, this question is not applicable.*

   *This should include a thorough description of their leadership role(s) in the study. Are local researchers named in the author list or the acknowledgements, or are they not mentioned at all (and, if not, why)? Please also describe the involvement of early career researchers based in the location of the study. Some of this information might be repeated from the Contributors section in the manuscript. Note: we adhere to [ICMJE authorship criteria](#) when deciding who should be named on a paper.*

| |
|---|
| **a) Study design:**<br>Local researchers from Malawi and Tanzania are co-applicants on our funding application and have contributed to study design from project conception. |
| **b) Clinical study processes:**<br>Local researchers from Malawi and Tanzania were involved with or led on all local study processes, such as participant screening, recruitment, data collection. |
| **c) Data interpretation:**<br>Within the consortium, we supported three PhD students to develop the analytical skills required to formulate the results of this manuscript, under supervision from the study statistician and senior academics within the partnership.<br>Research partners collaborated in interpreting study data through regular online meetings to present and discuss data to the Multlink consortium. In addition, we held an in-person meeting in January 2024 in Dar es Salaam, Tanzania to discuss and interpret the data.<br>Members of the Multilink Consortium had access to data hosted by the Malawi-Liverpool-Wellcome Progamme data portal, in line with the Data Management Plan. |
| **d) Manuscript preparation:**<br>PhD students within the Multilink consortium led the writing – Stephen Spencer (MLW, Malawi; LSTM, UK; clinical and demographic sections); Nateiya Yongolo (Moshi, Tanzania; health economic sections) – working directly with their PhD supervisors within the Multilink consortium. Senior authors on the manuscript also include local researchers. The manuscript was shared to the wider Multilink consortium partners for additional input. |

2. Were the data used in your study collected by authors named on the paper, or have they been extracted from a source such as a national survey? ie, is this a secondary analysis of data that were not collected by the authors of this paper. If the authors of this paper were not involved in data collection, how were data interpreted with sufficient contextual knowledge?

   The Lancet Global Health *believe contextual understanding is crucial for informed data analysis and interpretation.*

| |
|---|
| Data used in this study was collected by authors named in the paper and/or recognised by name within the Multilink consortium (listed within the manuscript) |

3. How was funding used to remunerate and enhance the skills of researchers and institutions based in the area(s) of study? And how was funding used to improve research infrastructure in the area of study?

   *Potentially effective investments into long-term skills and opportunities within institutions could include training or mentorship in analytical techniques and manuscript writing, opportunities to lead all or specific aspects of the study, financial remuneration rather than requiring volunteers, and other professional development and educational opportunities.*

   *Improvements to research infrastructure could be funding of extended trial designs (such as platform trials) and use of master protocols to enable these designs, establishment of long-term contracts for research staff, building research facilities, and local control of funding allocation.*

| |
|---|
| **Skills:**<br>All research activities in country were led, coordinated and conducted by staff from indigenous research institutions, with supportive collaboration from individuals and partners from other countries (e.g. US and UK) whilst also prioritising South:South (Malawi/Tanzania) knowledge exchange and scientific collaboration.<br>Research funding has been used to employ research teams, inclusive of funding four PhD students, two in Malawi and two in Tanzania.<br>Formal research training through PhD training is offered to four LMIC PhD researchers, who are listed as authors or collaborators within the manuscript.<br>We proactively sought out professional and skills development opportunities for early career scientists throughout the project – e.g. through co-authorship, co-analysis, analysis workshops and inter-country working groups (e.g. on data management, quant and qual data analysis). |
| **Research infrastructure:**<br>This project involves making enhanced commercially available CE marked diagnostic tests available in the four collaborating hospital sites.<br>All staff were paid for their contribution to the project (there were no unpaid volunteers) and participants were remunerated according to the schedule established by relevant indigenous research institutions. Funding for this research included funding research infrastructure for the local institutions to enhance their capacity in supporting the study in areas on data management, laboratory and ethical standards. Data management was led by the Data Management Team at MLW in Malawi.<br>All partners had control of their own funding allocation (within funder rules). |

4. How did you safeguard the researchers who implemented the study?

   *Please describe how you guaranteed safe working conditions for study staff, including provision of appropriate personal protective equipment, protection from violence, and prevention of overworking.*

| |
|---|
| |

All researchers are qualified professionals. The researchers attended safeguarding training and observed safeguarding policy procedures as per Liverpool School of Tropical Medicine when facilitating delivery of this project. All researchers were also GCP trained. Participants information sheets included information about safeguarding and contact details in case of reporting safeguarding issues

*<u>Benefits to the communities and regions of study</u>*

5. How does the study address the research and policy priorities of its location?

*How were the local priorities determined and then used to inform the research question? Who decided which priorities to take forward? Which elements of the study address those priorities?*

Prior to the start of the study, local priorities were determined at two levels:
Policy level - the Directorate of the Chronic Diseases in the MOH has been involved and supported application of this research funding. Subsequent presentations at the MOH technical working group identified prioritized for the research

Service delivery level - we engaged HCW, groups of patients and their caregivers, and community leaders (through established community advisory boards in the local institutions) in discussing their priorities in management of multimorbidity hence informing the design of the study

Multimorbidity, defined as the coexistence of two or more chronic diseases, is an increasing problem worldwide. In sub-Saharan Africa, the combination of high burden infectious disease and rising rates of non-communicable disease (NCD) is especially challenging. As people live longer, multimorbidity is increasingly common, often accelerated by HIV and the drugs required to treat it. High barriers to healthcare cause patients to delay seeking treatment until they are severely ill. During emergency assessments, doctors often focus on the primary presenting problem. This can be efficient, but misses an important opportunity in those with multimorbidity. Failing to identify and treat multimorbidity leads to frequent hospital readmissions, disability, death, and increased out-of-pocket expenses.

6. How will research products be shared in the community of study?

*For instance, will you be providing written or oral layperson summaries for non-academic information sharing? Will study data be made available to institutions in the region(s) of study?* The Lancet Global Health *encourages authors to translate the summary (abstract) into relevant languages after paper editing; do you intend to translate your summary?*

Translated abstracts will be provided with the published manuscript.
Data are being presented via to local stakeholders in each study site. For example, at local research dissemination conferences (such as the annual Kamuzu University of Health Sciences (KUHeS) Research Dissemination Conference. We have and are continuing to present our work directly to health-care-workers in the four hospitals where the study occurred. We have also

presented our findings to Technical Working Groups in Ministries of Health. Presentations also include those to community advisory boards in local institutions including some patients and caregivers who participated in the study

7. How were individuals, communities, and environments protected from harm?

a) *How did you ensure that sensitive patient data was handled safely and respectfully? Was there any potential for stigma or discrimination against participants arising from any of the procedures or outcomes of the study?*

*We used participant identification numbers for all patients recruited on the study. These numbers were captured on their clinical research files, lab test, database, to prevent stigma and discrimination. Access to this information was limited to study members only with login detail provided upon approval. Documents that contained names i.e. consent forms, were separately stored away and unlinked from patient data.*

b) *Might any of the tests be experienced as invasive or culturally insensitive?*

*No. All tests that patients went through are recognised as part of the local health service delivery and part of routine care using CE marked devises. Due to resources limitations in the local settings, some of the test, though recognised, were not available in the hospitals*

c) *How did you determine that work was sensitive to traditions, restrictions, and considerations of all cultural and religious groups in the study population?*

*Across the four implementation sites, we instituted Community Advisory Boards (CAB), comprising local leaders to advise on the study to ensure it is sensitive to traditions and culture of local settings. The CABs met prior to the start of the meeting and every quarter. Additionally, local ethics research committees and respective hospital research committees/ management teams approved the study.*

d) *Were biowaste and radioactive waste disposed of in accordance with local laws?*

*All biowaste was disposed of in accordance with local laws. We did not work with radioactive waste in our study.*

e) *Were any structures built that would have impacted members of the community or the environment (such as handwashing facilities in a public space)? If so, how did you ensure that you had appropriate community buy-in?*

*Not applicable*

f) *How might the study have impacted existing health-care resources (such as staff workloads, use of equipment that is typically employed elsewhere, or reallocation of public funds)?*

*Our study nurses supported local clinical activities in study sites.*
*Clinicians in the local hospitals utilised some of the diagnostic tests and results for patient management especially diagnostic tests that are not available in the hospitals*

8. Finally, please provide the title (eg, Dr/Prof, Mr/Mrs/Ms/Mx), name, and email address of an author who can be contacted about this statement. This can be the corresponding author.

**Name:** Dr Stephen Spencer
**Email: stephen.spencer@lstmed.ac.uk**
